# Vancomycin-Resistant, Thermotolerant *Enterococcus faecalis* and *Enterococcus faecium* in Ready-to-Eat Beef Luncheon and Sausage

**DOI:** 10.3390/foods15173112

**Published:** 2026-09-01

**Authors:** Sara Abdelnaby Sallam, Mirela Imre, Samir Mohammed Abd-Elghany, Kálmán Imre, Khalid Ibrahim Sallam

**Affiliations:** 1 Department of Food Hygiene, Safety, and Technology, Faculty of Veterinary Medicine, Mansoura University, Mansoura 35516, Egypt; saraabdelnaby529@gmail.com (S.A.S.); drsamir@msn.edu.eg (S.M.A.-E.); 2 Faculty of Veterinary Medicine, University of Life Sciences “King Mihai I” from Timisoara, 300645 Timisoara, Romania; mirela.imre@usvt.ro

**Keywords:** *Enterococcus*, virulence genes, antimicrobial resistance, vancomycin-resistant, heat processing, ready-to-eat meat products

## Abstract

This study investigated the prevalence, antimicrobial resistance profiles, virulence determinants, and heat tolerance of *Enterococcus faecalis* and *Enterococcus faecium* isolated from ready-to-eat (RTE) beef luncheon and sausage in Egypt. A total of 150 samples were analyzed, yielding 514 *Enterococcus* isolates, with contamination detected in all samples. Molecular identification revealed a predominance of *E. faecium* (71.2%) over *E. faecalis* (28.8%). High frequencies of virulence genes were detected, particularly *gelE* (95.7%) and *ace* (60.7%), indicating strong pathogenic potential. Antimicrobial susceptibility testing demonstrated widespread multidrug resistance, with high resistance rates to β-lactams, tetracycline (94%), vancomycin (62.5%), and rifampin (91%). Notably, 100% of *E. faecalis* and *E. faecium* isolates were classified as multidrug-resistant, with some strains exhibiting extensive or pandrug resistance. Vancomycin resistance genes (*vanA* and *vanB*) were detected in the majority of isolates, with *vanA* being predominant. Heat treatment experiments showed minimal reduction in bacterial counts after microwave heating (8.2 vs. 8.9 log_10_ CFU/g of the control), although boiling of the cooked RTE sausage for 5 min achieved a significant (*p* < 0.01) but incomplete reduction in the *Enterococcus* count by 3.8 logs (5.1 vs. 8.9 log_10_ CFU/g), indicating notable thermotolerance. Surviving isolates retained resistance and virulence traits. These findings highlight RTE meat products collected from Mansoura city as potential reservoirs of thermotolerant, multidrug-resistant enterococci, raising a potential public health concern and emphasizing the need for improved food safety measures and further nationwide studies. Although direct epidemiological links between foodborne isolates and clinical infections were not established, the presence of such strains raises a potential public health concern and underscores the need for improved food safety measures and further epidemiological investigations.

## 1. Introduction

Enterococci are widespread Gram-positive lactic acid bacteria (LAB) that are part of the normal microbiota of the gastrointestinal tracts of humans and animals. As a result, they are often used as indicators of potential fecal contamination in meat products during slaughter. Additionally, these bacteria serve as starter cultures responsible for ripening and aroma development in certain traditional cheeses and sausages, particularly those produced in the Mediterranean region. They are also used as active strains in commercial probiotic products to promote human health [1]. Enterococci can survive pasteurization, a pH range of 4.6 to 9.9, and high salt concentrations (6.5% *w*/*v*), which explains their survival during the processing of cooked meat products and ready-to-eat foods. They also multiply during the fermentation of uncooked cured meats [2].

Most Enterococci are generally commensal and not pathogenic to humans. However, certain species can cause hospital-acquired infections in immunocompromised individuals, which are often difficult to treat, especially multidrug-resistant strains with intrinsic resistance to many antibiotics commonly used in medicine [3]. Currently, more than 60 *Enterococcus* species have been identified, with *Enterococcus faecalis* and *Enterococcus faecium* being the most frequently isolated from foods and responsible for serious nosocomial infections in humans [4]. Among the top 10 blood pathogens worldwide, *Enterococcus* species cause infections such as bacteremia, endocarditis, meningitis, and urinary tract infections. They can lead to serious, potentially fatal conditions, especially in immunocompromised hosts [5,6]. The first documented case of *Enterococcus* infection was in 1899, resulting in acute endocarditis. The pathogenicity of Enterococci is linked to virulence factors such as *asa1* (aggregation substance), *cylA* (cytolysin), *esp* (enterococcal surface protein), *hyl* (hyaluronidase), and *gelE* (gelatinase) [7].

Research on the use of antimicrobials in food-producing animals has increased due to concerns about antimicrobial resistance (AMR). Understanding how resistant bacteria are transmitted through meat production and retailing is vital for devising strategies to reduce AMR, an escalating public health threat [8]. *Enterococcus* spp. can resist many antibiotics intrinsically or through horizontal gene transfer via mobile genetic elements such as plasmids and transposons, or through chromosomal mutations. Recently, vancomycin resistance in enterococci has garnered attention because this glycopeptide antibiotic is often used as a last resort for multidrug-resistant infections [9]. Vancomycin-resistant *E. faecium* has been designated a high priority for developing new treatments among about 12 antibiotic-resistant pathogens posing significant threats to human health.

Vancomycin resistance can be mediated intrinsically by the *vanC* gene, which confers low-level, non-transferable resistance. Acquired resistance involves eight van genes (*vanA*, *vanB*, *vanD*, *vanE*, *vanG*, *vanL*, *vanM*, and *vanN*), with *vanA* and *vanB* being the most common and clinically significant [10,11]. Strains with *vanA* or *vanB* genes have been identified in clinical cases, outbreaks, and across food animals worldwide.

Although direct links between human infections and consumption or handling of VRE-contaminated foods are limited, the presence of VRE (Vancomycin-Resistant Enterococci) strains carrying resistance genes in foodstuffs is a major public health concern due to their potential to spread resistance genes to other strains within the food chain [11,12,13]. Studying resistance genes in foods of animal origin is essential for understanding and controlling antimicrobial resistance. VRE studies in meat have been conducted globally, but no such research has been performed in Egypt. Therefore, the current study aims to determine the prevalence and resistance patterns of VRE in popular ready-to-eat meat products such as sausage and luncheon in Egypt, and to investigate the presence of resistance and virulence genes.

## 2. Materials and Methods

### 2.1. Sample Collection

One hundred and fifty ready-to-eat meat product samples (75 each of beef luncheon and sausage sandwiches) were randomly collected over ten sampling occasions between July 2023 and April 2024 from 25 different restaurants and supermarkets distributed across Mansoura city, Egypt. To capture variation in retail practices, the selected establishments included different levels of retail outlets, ranging from small/local establishments to medium-sized and larger restaurants and supermarkets, without restriction to a specific processing source or commercial brand. Importantly, the selected establishments were supplied by different manufacturers, ensuring diversity in the sources of the products sampled. The outlets were randomly selected within these different retail levels, without restriction to a specific processing source or commercial brand. On each sampling occasion, 5 restaurants and supermarkets were visited, and 3 samples of each luncheon and sausage sandwich were collected from each restaurant. Each sample was packaged individually in a sterile, impermeable polyethylene bag, labeled, and transferred within 1 h to the laboratory in an icebox at approximately 4 °C for bacteriological analyses.

### 2.2. Isolation and Identification of Enterococcus

The test portion of 25 g of meat products was excised aseptically with a sterile scalpel for each sample and transferred to a sterile homogenizer flask containing 225 mL of sterile buffered peptone water (Oxoid™ CM0509; Oxoid Ltd., Basingstoke, UK) and homogenized for 1 min in a stomacher (Seward Medical Ltd., Worthing, West Sussex, UK). The homogenate was incubated at 37 °C for 24 h. A volume of 1 mL was added to 10 mL of Man, Rogosa & Sharpe broth (MRS, Oxoid, Basingstoke, UK) and incubated at 37 °C for 24 h. A loopful from each enriched broth was spread on Bile Esculin Azide agar (M493I; HiMedia Laboratories Pvt. Ltd., Mumbai, India) plates and incubated at 37 °C for 24 h. Three to four typical colonies (small, dark brown to black colonies surrounded by a distinct black halo) of presumptive *Enterococcus* were subcultured onto BD Difco™ Brain Heart Infusion Agar (Thermo Fisher Scientific Inc., Waltham, MA, USA) slopes and incubated at 37 °C for 24 h for further biochemical identification through the following tests: Gram stain (Gram-positive cocci); catalase (−); growth (+) at 44 ± 1 °C, growth (+) in BHI containing 6.5% NaCl at 37 ± 1 °C for 48 h, growth at 10 °C and 45 °C, growth at pH 9.6, as well as growth and esculin hydrolysis on bile-esculin agar. A total of 514 colonies were obtained, and all selected colonies were biochemically confirmed as presumptive *Enterococcus* isolates. No isolate failed the biochemical confirmation criteria. All 514 biochemically confirmed isolates were subsequently subjected to *sodA*-PCR for molecular confirmation and species-level identification Biochemically confirmed *Enterococcus* spp. Isolates were further identified by molecular characterization.

### 2.3. Molecular Characterization of Enterococcus Isolates

The chromosomal DNA of each suspected *Enterococcus* isolate was extracted using the QIAamp DNA Mini Purification kit (QIAGEN GmbH, Hilden, Germany), according to the manufacturer’s instructions. Genomic DNA of the *E. faecalis* ATCC 29212 strain and the *E. faecium* ATCC 19434 strain served as positive control reference strains; meanwhile, *Escherichia coli* K12DH5α served as negative control reference strains for PCR analyses. All reference strains were obtained from the National Research Centre (NRC), Dokki, Cairo, Egypt, to assess the presence or absence of marker, virulence, and antimicrobial resistance genes.

Phenotypically identified *Enterococcus* isolates (*n* = 514), recovered from examined RTE meat product samples, were molecularly identified by using the polymerase chain reaction assays, based on the presence of *sodA* genes encoding manganese superoxide dismutase in *E. faecalis* and *E. faecium*. The identified strains were then tested for the presence of the gelatinase (*gelE*) and collagen-binding protein (*ace*) virulence genes. The *Enterococcus* isolates exhibiting phenotypic resistance to vancomycin (*n* = 321) by the agar dilution method were further analyzed by polymerase chain reaction (PCR) to detect vancomycin-resistance genes (*vanA* and *vanB*). The oligonucleotide primer sequences used to amplify marker, virulence, and antimicrobial resistance genes of *Enterococcus faecalis* and *E. faecium* isolates, along with the amplicon sizes and PCR conditions, were previously described [14].

The PCR amplification of the target genes was performed using a SimpliAmp™ Thermal Cycler (Thermo Fisher Scientific Inc, Waltham, MA, USA) in a 25-μL volume, which included 2 μL of *Enterococcus* genomic DNA template, 1 μL of each forward and reverse primer at 10 pmol (Sigma-Aldrich Co., St. Louis, MO, USA), 12.5 μL of DreamTaq Green Master Mix (Thermo Scientific, St. Leon-Rot, Germany), and 8.5 μL of sterile DNase/RNase-free distilled water. After an initial denaturation at 94 °C for 4 min, 35 cycles were performed (94 °C for 45 s, 56 °C for 60 s, 72 °C for 60 s), followed by a final extension at 72 °C for 7 min. The PCR products from each reaction were separated by loading 8 µL aliquots onto a 1.5% agarose gel (Cleaver Scientific–Horizontal Electrophoresis System; Cleaver Scientific Ltd., Rugby, UK) and running for 30 min at 100 V, then stained for 25 min in a 1% ethidium bromide solution (Sigma-Aldrich Co., St. Louis, MO, USA). The separated PCR products were visualized and photographed under a UV transilluminator. A GeneRuler 100 bp DNA Ladder (Thermo Fisher Scientific Inc.) served as a molecular size marker to estimate the sizes of the PCR products.

### 2.4. Antimicrobial Susceptibility Testing of Isolated E. faecalis and E. faecium

The antimicrobial susceptibility of 514 *Enterococcus* isolates, comprising 148 *E. faecalis* and 366 *E. faecium*, was evaluated against seventeen antimicrobial agents representing twelve antibiotic classes based on the standard medical/CLSI classification. Susceptibility testing was carried out using the Kirby–Bauer disk diffusion technique on Mueller–Hinton agar (MH; CM0337; Oxoid Ltd., Basingstoke, UK) in accordance with the Clinical and Laboratory Standards Institute (CLSI) guidelines [15]. Vancomycin susceptibility was determined using the agar dilution method. According to CLSI criteria [15], *Enterococcus* isolates with minimum inhibitory concentration (MIC) values ≤ 4 µg/mL were classified as susceptible, while those with MICs of 8–16 µg/mL were classified as intermediate, and isolates with MICs ≥ 32 µg/mL as vancomycin-resistant.

The antibiotics, represented multiple classes, were tested using commercial antibiotic disks (Oxoid Ltd., Basingstoke, UK). The antibiotics and disk concentrations used including the following: gentamicin (CN, 10 μg); nalidixic acid (NA, 30 μg), ciprofloxacin (CIP, 5 μg), and levofloxacin (LEV, 5 μg); penicillin G (P, 50 μg; Amoxicillin—10 μg); trimethoprim–sulfamethoxazole (SXT, 25 μg); cephalexin (CL, 30 μg), cefaclor (CEC, 30 μg), and cefepime (FEP, 30 μg); tetracycline (TE, 30 μg); linezolid (LZD, 10 μg); imipenem (IPM, 10 μg); chloramphenicol (C, 30 μg); rifampin (RA, 30/10 μg); and azithromycin (AZM, 15 μg).

Linezolid susceptibility was initially assessed by disk diffusion and confirmed by MIC determination, with MIC breakpoints of ≥8 µg/mL considered resistant.

The multiple antibiotic resistance (MAR) index for each *Enterococcus* isolate was determined following the method described by Singh et al. [16], calculated as the ratio of the number of antibiotics to which the isolate was resistant to the total number of antibiotics examined. The MAR index values exceeding 0.2 suggest excessive or inappropriate antimicrobial usage, while values greater than 0.4 are commonly associated with isolates of human fecal origin [17].

### 2.5. Effect of Extra Cooking of RTE Sausage and Luncheon Rolls on the Survival of Enterococcus

Three independent experimental trials were conducted to evaluate the effect of additional heat treatment on naturally occurring *Enterococcus* spp. in RTE meat products. Samples of sausage sandwiches and luncheon rolls were randomly obtained from various restaurants and retail markets in Mansoura City, Egypt.

In each trial, pre-cooked beef sausage samples (*n* = 9; 30 g each; 2 cm diameter) were randomly allocated into three groups (*n* = 3 per group): (i) untreated control, (ii) boiling treatment, and (iii) microwave treatment using an LG 42 Liters Microwave Oven with Grill at 220 V, 2450 MHz, 1200 W for 5 min (MH8265DIS; LG Electronics, Seoul, Republic of Korea). For the boiling treatment, the pre-cooked sausage samples were completely immersed in boiling water. The 5-min treatment period was counted from the beginning of boiling, during which the internal core temperature of the sausage samples reached approximately 85 °C. Following treatment, samples were aseptically removed and processed for enumeration of viable enterococci.

Similarly, beef luncheon rolls (*n* = 9; 250 g each; approximately 7 cm in diameter) were randomly divided into three groups (*n* = 3 per group): (i) untreated control, (ii) microwave treatment for 5 min, and (iii) microwave treatment for 10 min under the same conditions. The internal temperature of the samples was not measured during or immediately following microwave treatment.

Microbiological analysis was performed before and after heat treatments. For each sample, 25 g was aseptically homogenized in 225 mL of buffered peptone water, and appropriate serial dilutions (10^−2^ to 10^−8^) were prepared. Aliquots (0.1 mL) were spread onto Bile Esculin Azide agar (M493I; HiMedia Laboratories Pvt. Ltd) plates and incubated at 37 °C for 24 h. The 10^−4^ dilution was used for sausage samples, and the 10^−3^ dilution was used for luncheon samples, as these dilutions yielded countable colonies (25–250 CFU/plate) for accurate enumeration. Presumptive *Enterococcus* colonies were enumerated, and results were expressed as log_10_ CFU/g. For statistical purposes, each individual sample was treated as an experimental unit, and data from the three independent trials (*n* = 9 per treatment) were used for analysis.

**Figure 1:**
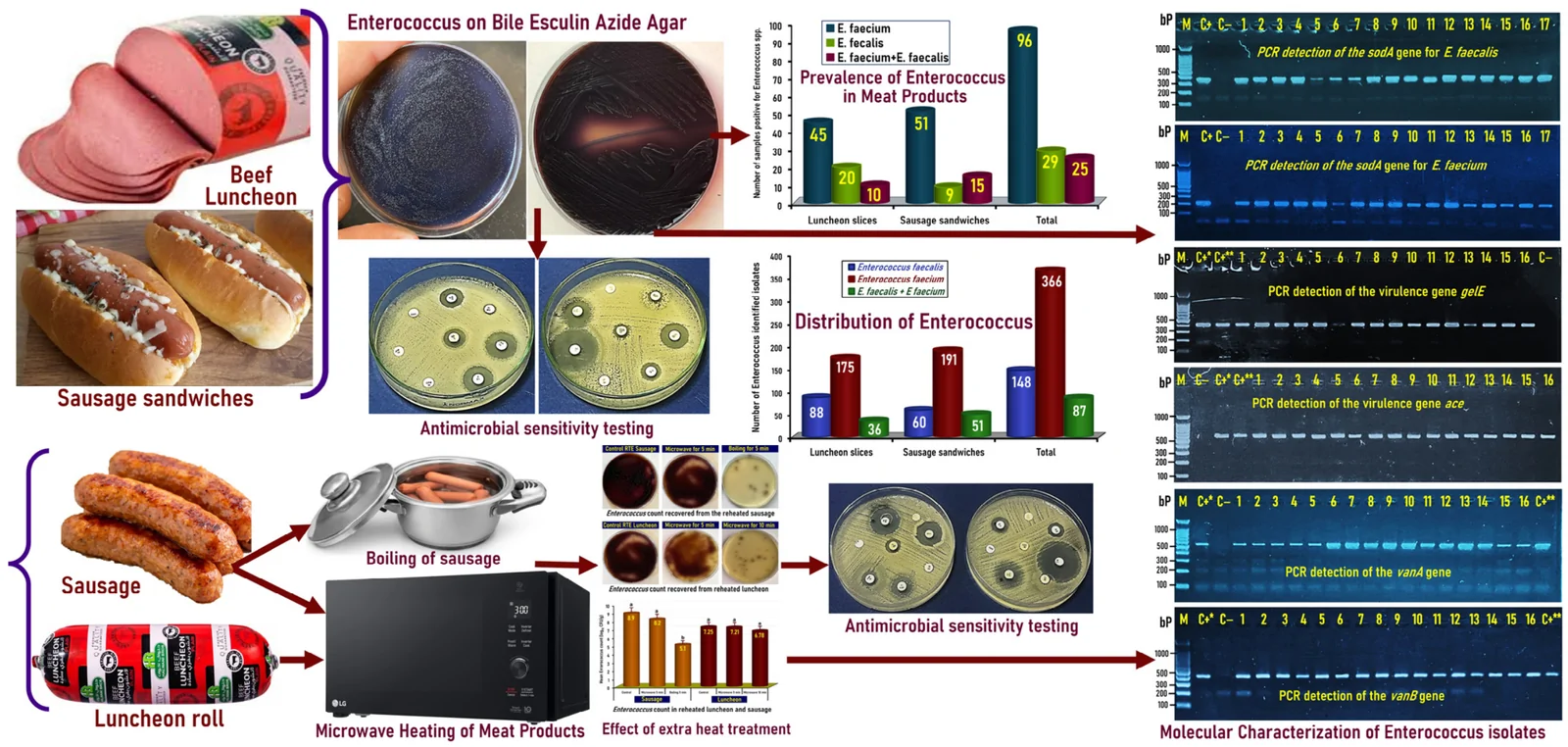
Graphical abstract illustrates the experimental workflow and major findings of the present study. Ready-to-eat (RTE) meat products, including beef luncheon, sausage sandwiches, sausage, and luncheon rolls, were examined for the prevalence and distribution of *Enterococcus faecalis* and *Enterococcus faecium*. Isolation and identification were performed using bile esculin azide agar, followed by antimicrobial susceptibility testing of the recovered isolates. Molecular characterization was conducted by PCR targeting species-specific (*sodA*) and virulence-associated (*gelE* and *ace*) genes, as well as vancomycin resistance genes (*vanA* and *vanB*). Positive controls were *E. faecalis* ATCC 29212 (C+*) and *E. faecium* ATCC 19434 (C+**), whereas *Escherichia coli* K-12 DH5α served as the negative control (C−). The effect of additional heat treatment (boiling and microwave heating) on the survival of *Enterococcus* spp. in RTE meat products was also evaluated.

A schematic overview of the experimental design and procedures employed in the present study is presented in the graphical abstract (Figure 1). All procedures were performed in compliance with relevant laws and institutional guidelines approved by the Mansoura University Animal Care and Use Committee (MU-ACUC) under the proposal code of “VM.MS.23.04.57”.

### 2.6. Statistical Analysis

All experiments were conducted in triplicate, and the *Enterococcus* counts (log_10_ CFU/g) were expressed as mean ± SD. The normality of data distribution and homogeneity of variances were assessed using the Shapiro–Wilk and Levene’s tests, respectively. One-way analysis of variance (ANOVA) followed by Tukey’s post hoc test was applied to estimate the differences between control and heat-treated sausage samples (before heating, after microwave heating, after boiling) as well as between the three luncheon groups (control group, microwave heating for 5 min, and microwave heating for 10 min). Statistical analyses were performed using IBM SPSS Statistics for Windows, Version 26.0 (IBM Corp., Armonk, NY, USA). Differences were considered statistically significant at *p* < 0.05.

## 3. Results and Discussion

### 3.1. Prevalence and Distribution of Enterococcus Species Isolated from Luncheon Slices and Sausage Sandwiches

**Figure 2:**
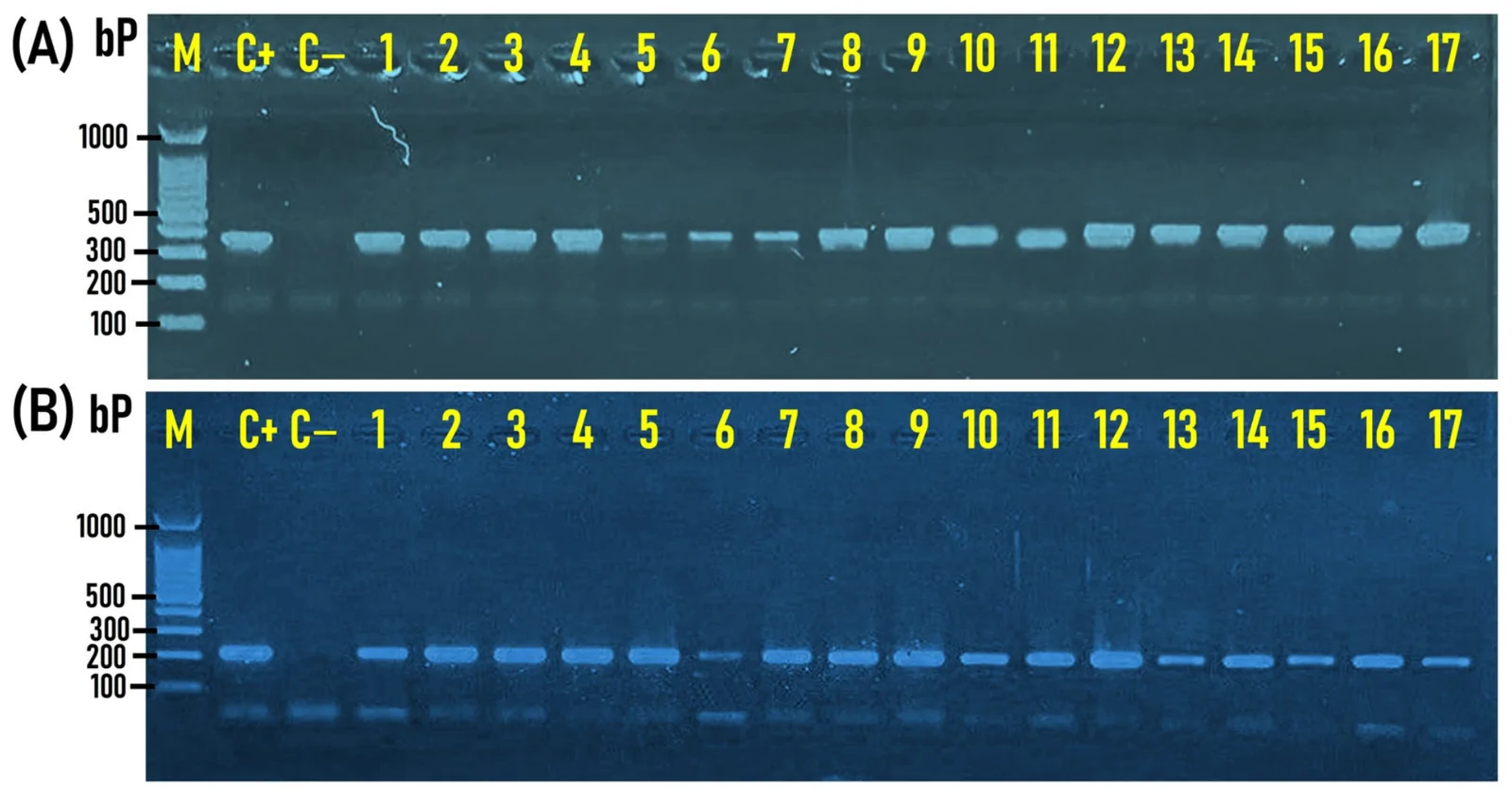
Molecular identification of *Enterococcus faecalis* and *Enterococcus faecium*. Agarose gel electrophoresis of PCR products targeting the *sodA* gene is shown. Lanes 1–17 represent isolates obtained from ready-to-eat (RTE) meat samples. Amplicons were observed at the expected sizes of 360 bp for *E. faecalis* (panel (**A**)) and 215 bp for *E. faecium* (panel (**B**)). M: 100 bp DNA ladder. Positive controls (C+) included *E. faecalis* ATCC 29212 (**A**) and *E. faecium* ATCC 19434 (**B**), while *Escherichia coli* K-12 DH5α was used as the negative control (C−). PCR products (8 µL) were resolved on a 1.5% agarose gel and visualized under ultraviolet illumination.

In this study, all (100%, 150/150) of the ready-to-eat meat samples tested, including sausage sandwiches (75/75) and luncheon slices (75/75), were positive for *Enterococcus* species. The biochemically identified 514 *Enterococcus* isolates (251 from sausage sandwiches and 263 from luncheon slices) were further confirmed and differentiated into *Enterococcus faecalis* and *Enterococcus faecium* using PCR for the detection of superoxide dismutase; *sodA* (*E. faecalis*) and *sodA* (*E. faecium*) marker genes, which were detected at the anticipated molecular sizes of 360 bp for *E. faecalis* (Figure 2A) and 215 bp for *E. faecium* (Figure 2B). Of the 514 isolates tested, 148 (28.79%) were molecularly verified as *E. faecalis*, and 366 (71.20%) were verified as *E. faecium*.

The 100% recovery of *Enterococcus* spp. should be interpreted with consideration of the enrichment-based isolation method used. Enrichment may enhance recovery of low-level contamination and consequently increase apparent prevalence. Thus, the present finding reflects recovery of culturable enterococci rather than uniformly high bacterial loads. Quantitative or direct-plating methods would be needed to determine the actual contamination levels.

Nearly similar to our findings, Aslam et al. [18] revealed that 96.3% (129/134) of tested retail beef samples in Canada were positive for *Enterococcus*. Additionally, Klibi et al. found that 80% (84/105) of meat samples recovered from Tunisian markets were contaminated with *Enterococcus* spp. [19]. On the other hand, lower prevalence rates were recorded for *Enterococcus* in meat and meat products tested worldwide. For instance, prevalence rates of 61.4% (35/57) [20], 60% (18/30) [21], 56.7% (34/60) [22], 45% (45/100) [23], and 30.8% (70/227) [24] were reported in meat and meat products tested in Poland, Brazil, Egypt, Canada, and Italy, respectively.

**Figure 3:**
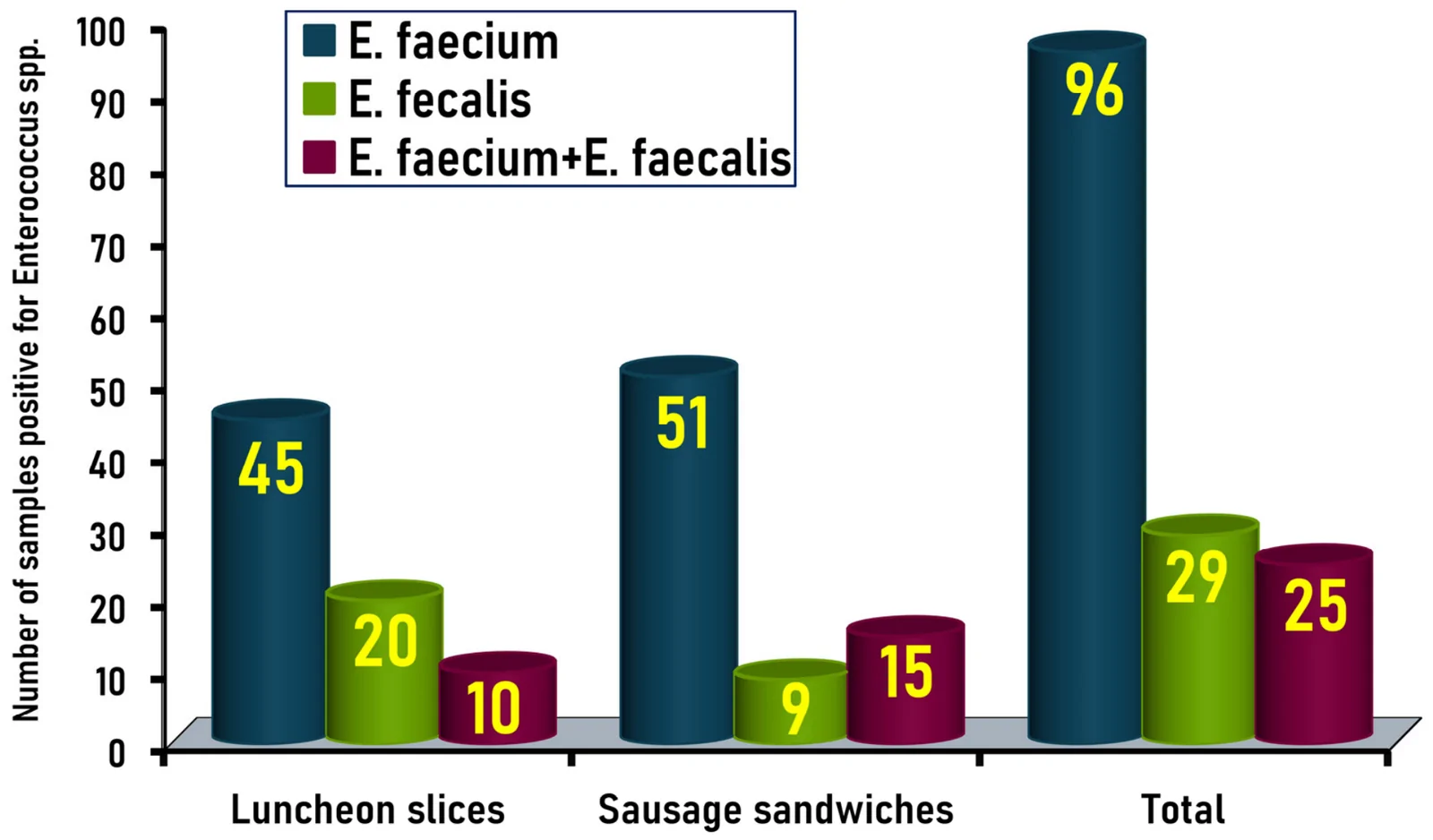
Prevalence of *Enterococcus faecalis* and *Enterococcus faecium* among ready-to-eat (RTE) luncheon and sausage sandwich samples, indicating the number of samples positive for each species separately and in combination.

The molecular characterization of the isolated *Enterococcus* spp. revealed that 64% (96/150), 19.33% (29/150), and 16.66% (25/150) of tested meat products were positive for *E. faecalis*, *E. faecium*, and co-occurrence of both *E. faecalis* and *E. faecium*, respectively (Figure 3). The distribution of *Enterococcus* on the product level indicated that 60% (45/75), 26.66% (20/75), and 13.33% (10/75) of tested luncheon slices; and 68% (51/75), 12% (9/75), and 20% (15/75) of tested sausage sandwiches, were positive for *E. faecalis*, *E. faecium*, and co-occurrence of both *E. faecalis* and *E. faecium*, respectively (Figure 3).

The high contamination rates of ready-to-eat (RTE) meat products with *Enterococcus* spp. in the present study represent a major public health concern because these foods are consumed without further heating. These bacteria, particularly *E. faecalis* and *E. faecium*, are highly heat-resistant and can survive processing, persist in harsh conditions, and grow during fermentation. Contamination is mainly linked to inadequate hygiene during processing, contaminated equipment or additives, improper handling, post-processing exposure, and inadequate storage conditions [2].

### 3.2. Distribution of Enterococcus Isolates (n = 514) Among Luncheon Slices and Sausage Sandwiches

**Figure 4:**
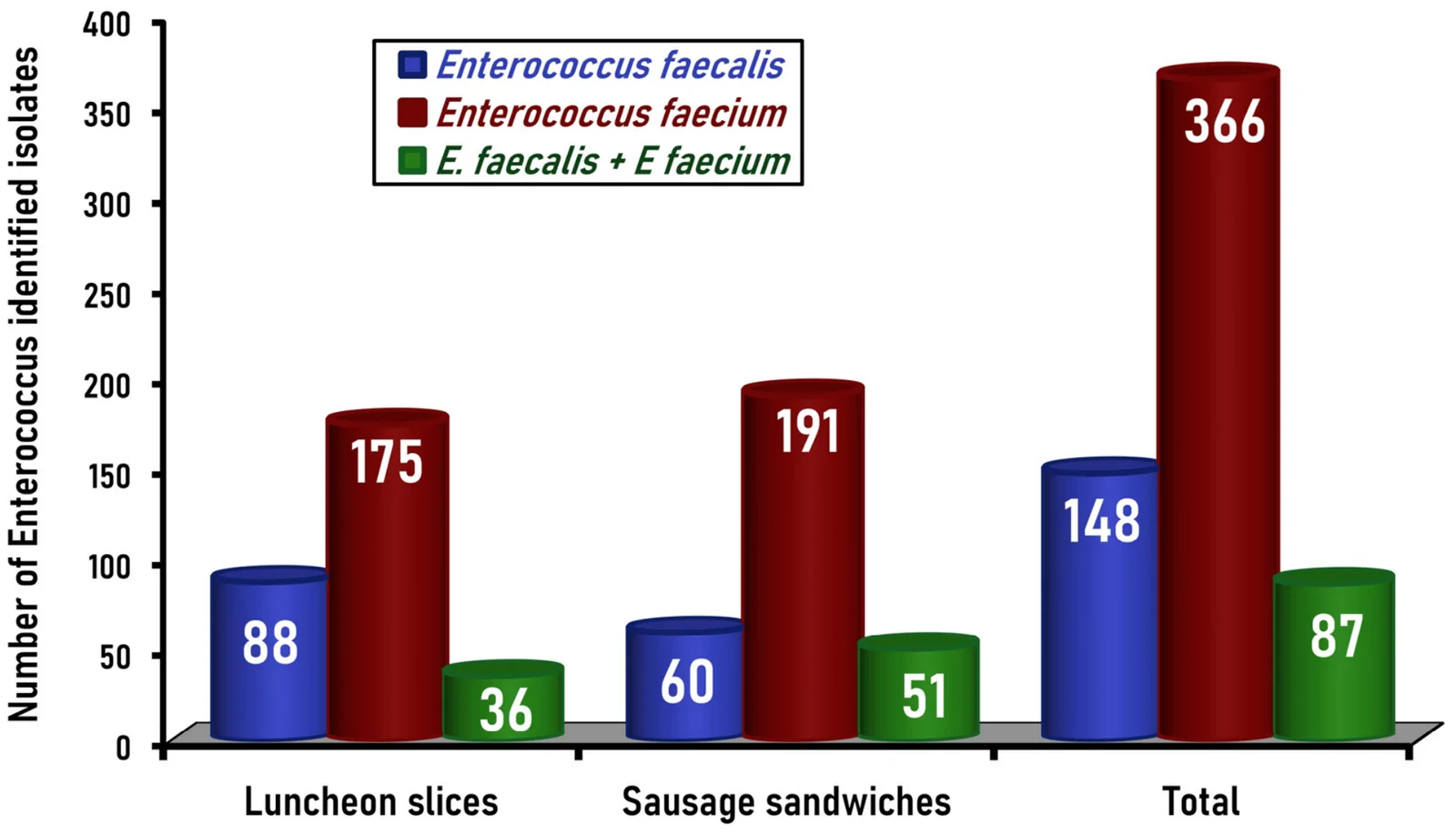
Distribution of *Enterococcus faecalis* isolates (*n* = 148) and *Enterococcus faecium* isolates (*n* = 366) recovered from luncheon slices and sausage sandwiches. Blue bars represent *E. faecalis*; orange bars represent *E. faecium*. Green bars represent the total number of *Enterococcus* isolates recovered from each product type (i.e., the sum of *E. faecalis* and *E. faecium* isolates combined). Data are presented as the number of isolates per species and product type. The total number of *Enterococcus* isolates recovered from luncheon slices was 263 (88 *E. faecalis* + 175 *E. faecium*), while 251 isolates were recovered from sausage sandwiches (60 *E. faecalis* + 191 *E. faecium*).

Of the 514 molecularly identified *Enterococcus* isolates, 148 (28.8%) were confirmed as *E. faecalis* and 366 (71.2%) were verified as *E. faecium*. The distribution of *Enterococcus* isolates (*n* = 514) among the RTE meat products examined indicated that 88 (59.46%) of *E. faecalis* isolates were recovered from 20 luncheon slices and 175 (47.81%) of *E. faecium* isolates were recovered from 45 luncheon slices; while 60 (40.54%) and 191 (52.19%) of the *E. faecalis* and *E. faecium* isolates were recovered from sausage sandwich samples, respectively (Figure 4). On the other hand, the 10 luncheon slices and the 15 sausage samples that were positive for mixed contamination by both *E. faecalis* and *E. faecium* yielded 36 and 51 *Enterococcus* isolates. Notably, *E. faecium* emerged as the most dominant species, followed by *E. faecalis*. This finding is in consistent with those reported by Valenzuela et al., who found that *E. faecium* was the most dominant species (64%, 16/25) isolated from foods of animal origin in Serbia followed by *E. faecalis* (36%, 9/25) [25], and also to that of Gomes et al., who reported that *E. faecium* was the most common species (61.7%, 37/60) obtained from meat products in Brazil, followed by *E. faecalis* (20%, 12/60) [21].

The prevalence of *E. faecium* in this study was higher than that reported by Pesavento et al., who estimated that 55.3% (21/38) of enterococcal isolates obtained from sausage in Italy were *E. faecium* [24]. Likewise, the prevalence of *E. faecium* was 44.8% (13/29) among isolated *Enterococci* from meat and fermented dry sausage in Canada [22]. Additionally, lower prevalence rates of 35.7% (25/70) [24] and 35.6% (16/45) [23] were reported for *E. faecium* in meat samples from Italy. Moreover, *Enterococcus faecium* was present in very low prevalence rates of 28.6% (77/269), 25% (30/119), 24.2% (44/182), 15.5% (11/71), and 2.3% (3/129) in meat samples tested in Botswana [26], Tunisia [19], Portugal [27], Slovenia [28], and Canada [18], respectively.

Globally, *E. faecium* has become one of the most troublesome pathogens that are responsible for several human diseases, including endocarditis and wound infections that are resistant to many drugs. Crucially, *E. faecium* exhibits significant resistance to many commonly used hospital antibiotics, which makes treatment of its infections more difficult [29].

Of the 514 *Enterococcus* isolates recovered from RTE meat tested in the current study, 148 (28.8%) were classified as *E. faecalis*. Several higher prevalence rates of *E. faecalis* in meat products were reported worldwide by several researchers, for example, 84.5% (60/71) in Slovenia [28], 73% (94/129) in Canada [18], 57.2% (154/269) in Botswana [26], and 51.7% (15/29) in Canada [22]. Furthermore, relatively higher recovery rates of *E. faecalis* at 36.8% of sausage and 41.8% of fermented meat products were reported in Italy [24] and Portugal [27], respectively.

*E. faecalis* is becoming more widely acknowledged as a major cause of both community- and hospital-acquired urinary tract infections, which may progress to severe conditions like bacteremia. It can also infect multiple body systems, including the skin, heart, bloodstream, and gastrointestinal tract. Its resistance to many antimicrobial drugs makes it a significant and challenging pathogen in healthcare settings [30].

### 3.3. Prevalence of Virulent Genes Among Enterococcus Species Isolated from Beef Luncheon and Sausage

**Figure 5:**
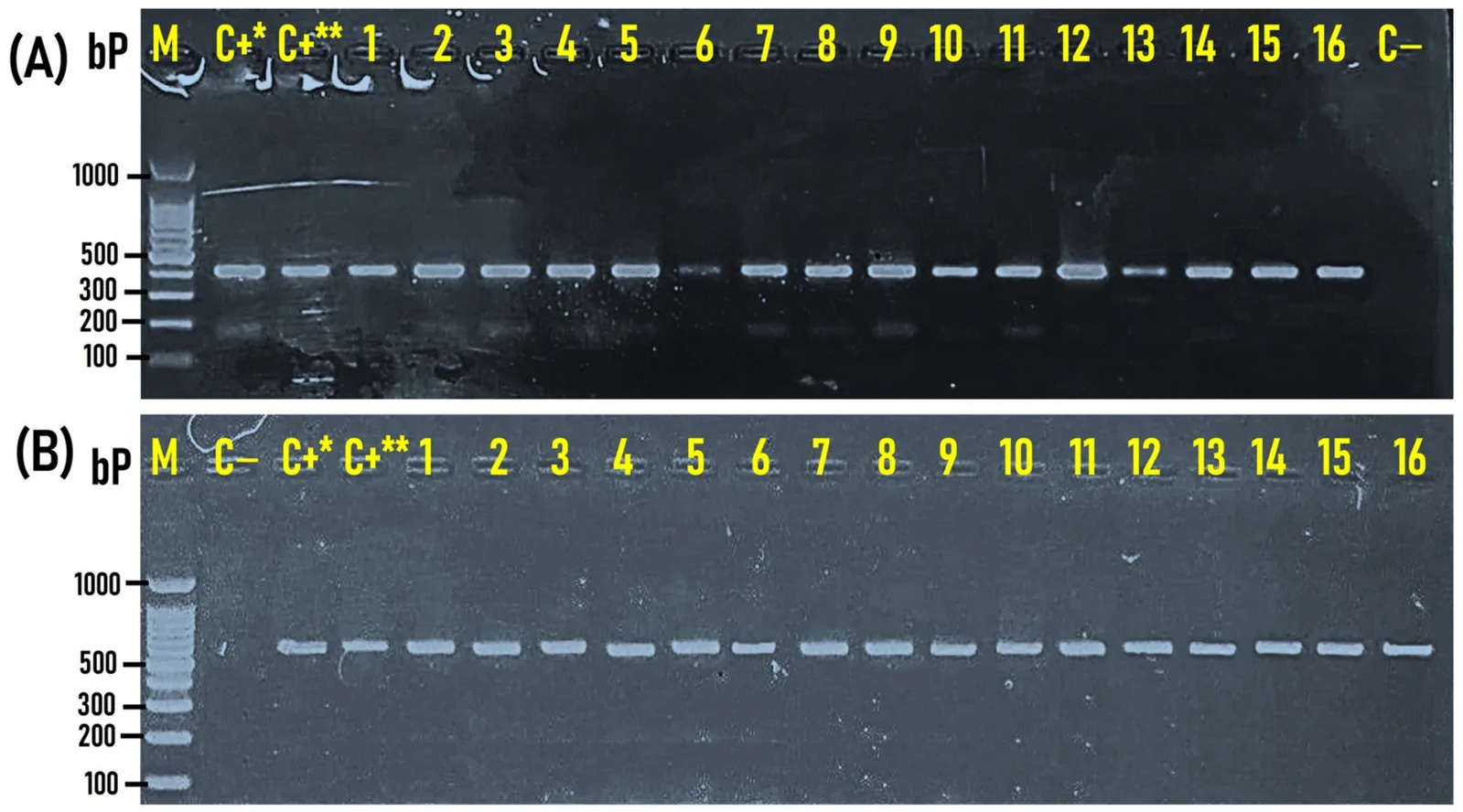
Molecular characterization of virulence-associated genes in *Enterococcus* isolates. Agarose gel electrophoresis of PCR amplicons targeting the *gelE* (**A**) and *ace* (**B**) genes is presented. Lanes 1–8 correspond to *Enterococcus faecalis* isolates, while lanes 9–16 represent *Enterococcus faecium* isolates recovered from ready-to-eat (RTE) luncheon and sausage sandwiches. Amplification products were observed at the expected sizes of 419 bp for the *gelE* gene (**A**) and 616 bp for the *ace* gene (**B**). M: 100 bp DNA ladder. Positive controls included *E. faecalis* ATCC 29212 (C+*) and *E. faecium* ATCC 19434 (C+**), whereas *Escherichia coli* K-12 DH5α served as the negative control (C−). PCR products (8 µL) were separated on a 1.5% agarose gel and visualized under ultraviolet illumination.

In this study, alongside the *sodA* gene, which was identified in all *Enterococcus* isolates (Table 1), PCR was used to further target the *gelE* and *ace* virulence genes encoding gelatinase and collagen-binding protein in *Enterococcus isolates,* which were observed at the expected molecular sizes of 419 bp (Figure 5A) and 616 bp (Figure 5B), respectively.

Gelatinase is an important virulence factor that helps the bacterium evade host immune defenses and contributes to its resistance against antimicrobial peptides and the innate immune system [31]. Gelatinase promotes biofilm formation in *Enterococcus* strains and degrades a variety of host proteins, including gelatin, elastin, collagen, hemoglobin, and other bioactive peptides. This highlights the crucial role of the *gelE* gene in virulence and host colonization [32].

The *gelE* gene was a predominant virulence gene among *Enterococcus* isolates recovered from luncheon slices and sausage sandwiches, with incidence rates of 91.6% (241/263) and 100% (251/251), respectively, and an overall prevalence of 95.7% (492/514) (Table 1). On the *Enterococcus* species basis, all (100%) of the 148 *E. faecalis* isolates and 94% (344/366) of *E. faecium* were positive for the *gelE* gene, with an overall prevalence of 95.7% (492/514) among all of the *Enterococcus* isolates (Table 1). This finding was nearly similar to that reported by Klibi et al., who found that 95.8% (46/48) of *Enterococcus* isolates obtained from meat samples from Tunisian markets had the *gelE* gene [19]. On the contrary, moderately lower *gelE* gene prevalence rates of 75% (75/100) of enterococcal isolates obtained from beef meat samples from butcher shops and supermarkets in Turkey [33] and 67.3% (33/49) of enterococcal isolates obtained from meat products in Brazil [21] were positive for the *gelE* gene. On the same context, very low *gelE* gene prevalence rates of 58.6% (17/29), 56% (79/141), 36% (9/25), and 35% (7/20) were reported in *Enterococcus* isolates collected from raw and fermented meat in Canada [22], retail red meat in Slovenia [28], dairy and meat products in Serbia [25], sausages in Portugal [34], respectively.

The high prevalence of the *gelE* gene (95.7%) detected in this study is consistent with previous reports and suggests that the genetic potential for gelatinase production is widespread among enterococci from RTE meat products. However, the detection of *gelE* by PCR indicates only the presence of the gene, not its functional expression. Gelatinase activity is regulated by the fsr quorum-sensing system, and its expression can be influenced by environmental conditions, growth phase, and regulatory mutations. Therefore, the actual pathogenic potential of these isolates may be lower than suggested by gene prevalence alone. While phenotypic assays for gelatinase activity were beyond the scope of this study, our findings highlight the need for future research that combines genotypic and phenotypic approaches to more accurately assess the virulence potential of foodborne enterococci.

The *ace* gene is strongly associated with severe clinical conditions, including bacteremia, urinary tract infections, and endocarditis. Moreover, Ace may facilitate immune evasion by reducing phagocytic clearance and impairing host immune recognition [35,36]. On the *Enterococcus* species basis, 56.8% (84/148) of *E. faecalis* isolates and 62.3% (228/366) of *E. faecium* isolates were positive for the *ace* gene (Table 1). The *ace* gene was detected in isolates recovered from luncheon and sausage, with high incidence rates of 64.5% (162/251) and 57% (150/263), respectively, and an overall prevalence of 60.7% (312/514) among *Enterococcus* isolates (Table 1). Similar results were determined among *E. faecalis* and *E. faecium* by Golob et al., who found the *ace* gene in 65.2% (92/141) of enterococcal isolates obtained from retail red meat in Slovenia [28]. In comparison, lower incidences of 42.9% (12/28) [22], 24.5% (12/49) [21], and 5% (1/20) [34] were reported for *ace* gene in *Enterococcus* isolates recovered from food tested in Canada, Brazil, and Portugal, respectively.

### 3.4. Antimicrobial Resistance Profile of Enterococcus Isolates (n = 514) from Beef Luncheon and Sausage

The Antimicrobial resistance profile of all verified *E. faecalis* and *E. faecium* isolates (*n* = 514) recovered from beef luncheon and sausage toward 17 antibiotics related to 12 antimicrobial classes tested is shown in Table 2.

Notably, all isolates (100%) were resistant to at least one β-lactam antibiotic. All *E. faecalis* (148/148) and *E. faecium* (366/366) isolates showed resistance to both cefepime and imipenem (Table 2). The universal cefepime nonsusceptibility aligned with the well-described intrinsic cephalosporin resistance of *Enterococcus* spp., which is attributable to low-affinity penicillin-binding proteins and other intrinsic mechanisms [37,38]. The present finding was substantially higher than the resistance rate of 22.2% (10/45) reported for *Enterococcus* isolates from meat products tested in Italy [23].

Large surveillance studies and recent meta-analyses reported broadly elevated β-lactam nonsusceptibility among clinical enterococcal isolates worldwide, although resistance proportions vary by species, geographic region, and clinical setting [30,39].

The universal non-susceptibility to cefepime is expected due to intrinsic cephalosporin resistance, mediated by PBPs with low beta-lactam affinity and cell-wall regulatory mechanisms [37]. This study indicates a substantial prevalence of cephalosporin resistance among the *Enterococcus* isolates. Specifically, 43% (221/514) of isolates were resistant to cephalexin, and an even higher proportion, 76.1% (391/514), exhibited resistance to cefaclor. The resistance rates reported here are markedly elevated. For instance, only 23.8% (55/231) of *Enterococcus* isolates from beef samples in Botswana were resistant to cephalothin, a first-generation cephalosporin similar to cephalexin [26].

In the present study, 100% of *E. faecium* (366/366) and 68.9% of *E. faecalis* (102/148) exhibited resistance to rifampin. This markedly high prevalence underscores a concerning level of rifampin resistance among enterococcal isolates from our sample set (Table 2). Earlier findings, though, show that variability across studies is evident. For instance, Barbosa et al. reported ~60% (109/182) rifampicin resistance among *Enterococcus* isolates from traditional fermented meat products in Portugal [27]. Meanwhile, Valenzuela et al. found 76% (19/25) resistance among *Enterococcus* isolates from foods of animal origin in Serbia [25]. On the other hand, a notably lower rifampin/resistance level (20%, 9/45) was reported in meat-derived enterococci from Italy [23]. The high resistance observed, particularly in *E. faecium*, raises public health concerns. Rifampin-resistant enterococci in food-associated isolates could facilitate the transfer of resistance genes through the food chain, potentially limiting treatment options for clinical infections.

In our dataset, 77.7% of *E. faecalis* and 93.7% of *E. faecium* exhibited resistance to penicillin, indicating substantial antimicrobial pressure or the circulation of resistant lineages in the sampled environment. In comparison, considerably lower resistance levels have been described elsewhere. For instance, Jahan et al. reported that only 23.1% of *E. faecium* and 0% of *E. faecalis* isolates from Canadian meat products were resistant to penicillin [22]. Similarly, Ribeiro et al. noted a 10% resistance rate among *E. faecalis* recovered from Portuguese sausage samples [34], while Yılmaz et al. found just 3% penicillin-resistant *E. faecalis* in minced beef [33], whereas Różańska et al. detected only 2.9% resistance among *E. faecalis* isolated from Polish beef samples [20]. Quite the reverse of our findings, Aslam et al. [18] identified no penicillin-resistant *E. faecalis* or *E. faecium* among 532 isolates from Canadian retail meat. This discrepancy may reflect differences in antimicrobial usage practices, geographical variation in resistance dissemination, or distinct sampling contexts and methodologies.

The presence of vancomycin-resistant enterococci (VRE) in meat products, particularly those consumed with minimal or no cooking, is alarming because vancomycin remains a last-resort antibiotic for serious enterococcal infections, raising significant concerns regarding foodborne transmission of VRE or their resistance genes to humans through the food chain, thereby complicating infection control and treatment. This study indicated that *E. faecalis* and *E. faecium* isolates exhibited high vancomycin resistance rates of 68.9% (102/148) and 59.8% (219/366), respectively, with an overall resistance rate of 62.5% (321/514) across both species (Table 2). Lower resistance rates against vancomycin were determined for *Enterococcus* species isolated from Swiss RTE meat products [40]. Similarly, research on meat preparations in Spain (including sausages, hamburgers, minced, and processed meats) reported VRE in 23.8% of samples, with 37.5% of poultry, 15.0% of pork, and 5.0% of beef samples [12]. While data remain limited for fully RTE meat across many regions, isolates from retail poultry meat in Egypt confirmed that animal-derived foods continue to serve as a reservoir for clinically relevant antimicrobial resistance [41].

The present work revealed high tetracycline resistance rates of 79.1% (117/148) and 100% (366/366) among *E. faecalis* and *E. faecium* isolates, respectively, with an overall resistance of 94% (483/514) across both species (Table 2). This is substantially higher than what many previous studies have reported in food and meat-derived enterococci. For example, in a large U.S. retail-meat surveillance spanning 2002–2014, resistance to tetracycline was found in 67.5% of *E. faecalis* and 53.7% of *E. faecium* isolates from retail meats [4]. Meanwhile, *E. faecium* isolates from processed pork products in a study from Korea exhibited much lower tetracycline resistance of 42.9% [42].

*Enterococcus* isolates from RTE meat products in this investigation showed a remarkably high level of 71.2% for Trimethoprim/Sulfamethoxazole resistance (Table 2). Similar reports of high resistance to sulfonamides have been reported for *Enterococcus* isolates from ready-to-eat meat products in Poland [43], although a much lower resistance rate of 21.2% has been reported for *Enterococcus* isolates from poultry farms in Uganda [44].

In this study, 45.5% (243/514) of *Enterococcus* isolates from ready-to-eat (RTE) meat products were resistant to azithromycin (Table 2), indicating substantial macrolide resistance. Similar findings have been reported elsewhere; Chajęcka-Wierzchowska et al. found 42.7% of RTE meat isolates resistant to erythromycin [43], while Pesavento et al. documented variable macrolide resistance in retail foods [24]. The high resistance in this study may reflect extensive macrolide use in food animals, and RTE meats could act as reservoirs for resistance genes transferable to human pathogens, highlighting the need for continued monitoring and control measures [43].

*Enterococcus* isolates from RTE meat products exhibited moderate resistance rates of 23% (118/514), 23.5% (121/514), and 29.8% (153/514) to ciprofloxacin, levofloxacin, and nalidixic acid, respectively (Table 2). Higher resistance rates were reported by Valenzuela et al., who observed that 72% (18/25) of *E. faecalis* and 56% (14/25) of *E. faecium* were resistant to ciprofloxacin and levofloxacin, respectively [25]. In contrast, several studies reported lower resistance rates in enterococcal isolates from meat products: 12% (12/100) in Turkey [33], 10.7% (3/28) in Canada [22], 1.4% (2/141) in Slovenia [28], 0% (0/35) in Poland [20], and 0% (0/94) in Canada [18] against ciprofloxacin. The moderate resistance observed in the current study highlighted a potential public health concern, as consumption of contaminated RTE meats could serve as a route for transferring resistant *Enterococcus* to humans, emphasizing the need for continuous surveillance and prudent antibiotic stewardship.

Notably, none (0%; 0/148) of the *E. faecalis* isolates and 34.2% (125/366) of the *E. faecium* isolates exhibited resistance to gentamicin (Table 2). Similar results have been reported in previous studies, where *E. faecalis* isolates from various meat samples showed 0% resistance to gentamicin [18,19,20]. In contrast, Ribeiro et al. observed a higher resistance rate, with 65% (13/20) of *E. faecalis* isolates being resistant [34]. For *E. faecium*, Klibi et al. reported a lower resistance rate of 3.3% (1/30) [19], while food isolates from Canada [22] and Slovenia [28] exhibited no resistance to gentamicin. The findings indicate that gentamicin remains highly effective against *E. faecalis*, with no resistance detected in the current study, consistent with previous reports from various meat sources. In contrast, *E. faecium* showed a higher resistance rate (34.2%), reflecting greater variability in susceptibility compared to *E. faecalis*. These results suggest that gentamicin remains a reliable option for treating *E. faecalis* infections, but the relatively higher resistance in *E. faecium* underscores the need for continued surveillance and prudent antibiotic use.

Linezolid is an oxazolidinone-class antibiotic that is regarded as a last-line treatment option for severe infections, particularly those caused by multidrug-resistant Gram-positive bacteria. Linezolid susceptibility was initially assessed by Kirby–Bauer disk diffusion and subsequently confirmed by agar dilution MIC determination. The results of both methods were fully concordant, with no discrepancies observed. In the present study, 85 out of 514 enterococcal isolates (16.5%) recovered from RTE meat products exhibited resistance to linezolid (Table 2). This prevalence is relatively higher than that reported by Elghaieb et al., who found approximately 5% linezolid resistance among enterococci isolated from retail meat and food-producing animals in Tunisia [45]. Conversely, a higher finding was documented in Poland by Zarzecka et al., who reported a 26.9% prevalence of linezolid-resistant enterococci in foods of animal origin [46]. The current findings revealed that 14% (72/514) of *Enterococcus* isolates were resistant to chloramphenicol (Table 2). Conversely, all *Enterococcus* isolates from red meat were susceptible to chloramphenicol [28]. In contrast, a higher resistance rate of 28.6% (10/35) was documented by Różańska et al. [20].

### 3.5. Multiple Antibiotic Resistance (MAR) Index and Categorization of Enterococcus Isolates According to Their Antibiotic Resistance Profiles

A considerable proportion of the isolates exhibited extensive drug resistance, as indicated by elevated MAR index values (0.705 to 0.882). Specifically, 8.1% of *E. faecalis* isolates from sausage sandwiches and 12.8% of *E. faecium* isolates from both food types were classified as extensively drug-resistant (Table 3). Alarmingly, the majority of isolates exhibited multidrug resistance (MDR; resistance to ≥3 antimicrobial classes), with 91.9% (136/148) of *E. faecalis* and 79.8% (292/366) of *E. faecium* meeting this criterion, underscoring the widespread dissemination of resistance across multiple antibiotic classes among foodborne enterococci (Table 3). Of particular concern, a subset of *E. faecium* isolates (7.4%; 27/366) recovered from sausage sandwiches exhibited resistance to all 12 antimicrobial classes tested, confirming the presence of pandrug-resistant strains (Table 3). The average MAR index of *Enterococcus* isolates tested was 0.461 and 0.600 for *E. faecalis* and *E. faecium*, respectively, with 97.27% (500/514) of *Enterococcus* isolates harboring a MAR index exceeding 0.2 (Table 3). A MAR index greater than 0.2 indicates the misuse of antimicrobials, whereas a value exceeding 0.4 generally points to human-related fecal contamination [17]. These findings underscore the potential public health risk associated with the consumption of ready-to-eat meat products contaminated with highly resistant *Enterococcus* strains.

Although pandrug-resistant *Enterococcus* isolates are less frequently reported, sporadic occurrences have been documented, underscoring the growing public health concern associated with limited therapeutic options [47]. Collectively, these findings, together with the high resistance levels observed in the present study, emphasize the potential role of ready-to-eat meat products as reservoirs for highly resistant *Enterococcus* strains and highlight the need for strengthened surveillance and antimicrobial stewardship across the food production chain.

**Table 3:** Classification of *Enterococcus* isolates (*n* = 514) based on their antibiotic resistance profile against seventeen antibiotics tested and their multiple antibiotic resistance (MAR) index.

*Enterococcus* spp.	Isolates Source	Antimicrobial Resistance Profile	Antimicrobial Resistance Categories/Classes	Number of Classes	MAR Index	* Classification of Strains	
						Type of Resistance	No. and (%)
*E. faecalis*	Sausage sandwiches (*n* = 12)	FEP, IPM, TE, RD, P, CEC, AMX, SXT, VA, CL, CIP, LEV, LZD, C	Cephalosporins (FEP, CEC, CL), Carbapenems (IPM), Tetracyclines (TE), Rifamycins (RD), Penicillins (P, AMX), Sulfonamides (SXT), Glycopeptides (VA), Quinolones (CIP, LEV), Oxazolidinones (LZD), Phenicols (C)	10	0.823	Extensively drug-resistant	12 (8.1%)
	Sausage sandwiches (*n* = 6)	FEP, IPM, TE, RD, P, CEC, SXT, AZM, CL, CIP, LEV, C	Cephalosporins (FEP, CEC, CL), Carbapenems (IPM), Tetracyclines (TE), Rifamycins (RD), Penicillins (P), Sulfonamides (SXT), Macrolides (AZM), Quinolones (CIP, LEV), Phenicols (C)	9	0.705	Multidrug-resistant	136 (91.9%)
	Luncheon slices (*n* = 21)	FEP, IPM, TE, RD, P, CEC, SXT, AZM, CL, NA, C	Cephalosporins (FEP, CEC, CL), Carbapenems (IPM), Tetracyclines (TE), Rifamycins (RD), Penicillins (P), Sulfonamides (SXT), Macrolides (AZM), Quinolones (NA), Phenicols (C)	9	0.647		
	Sausage sandwiches (*n* = 2)	FEP, IPM, TE, RD, P, SXT, AMX, AZM, CL, CIP, LEV, C	Cephalosporins (CL), Carbapenems (IPM), Tetracyclines (TE), Rifamycins (RD), Penicillins (P, AMX), Sulfonamides (SXT), Macrolides (AZM), Quinolones (CIP, LEV), Phenicols (C)	9	0.705		
	Luncheon slices (*n* = 3)	FEP, IPM, TE, RD, P, CEC, SXT, CL, NA, C	Cephalosporins (FEP, CEC, CL), Carbapenems (IPM), Tetracyclines (TE), Rifamycins (RD), Penicillins (P), Sulfonamides (SXT), Quinolones (NA), Phenicols (C)	8	0.588		
	Luncheon slices (*n* = 13)	FEP, IPM, TE, RD, P, CEC, AMX, SXT, VA, CL	Cephalosporins (FEP, CEC, CL), Carbapenems (IPM), Tetracyclines (TE), Rifamycins (RD), Penicillins (P, AMX), Sulfonamides (SXT), Glycopeptides (VA)	7	0.588		
	Luncheon slices (*n* = 1)	FEP, IPM, TE, RD, P, CEC, AMX, SXT, VA	Cephalosporins (FEP, CEC), Carbapenems (IPM), Tetracyclines (TE), Rifamycins (RD), Penicillins (P, AMX), Sulfonamides (SXT), Glycopeptides (VA)	7	0.529		
	Sausage sandwiches (*n* = 9)	FEP, IPM, TE, P, CEC, SXT, VA, RD	Cephalosporins (FEP, CEC), Carbapenems (IPM), Tetracyclines (TE), Penicillins (P), Sulfonamides (SXT), Glycopeptides (VA), Rifamycins (RD)	7	0.471		
	Sausage sandwiches (*n* = 15)	FEP, IPM, RD, P, CEC, SXT, VA	Cephalosporins (FEP, CEC), Carbapenems (IPM), Rifamycins (RD), Penicillins (P), Sulfonamides (SXT), Glycopeptides (VA)	6	0.412		
	Luncheon slices (*n* = 1)	FEP, IPM, TE, RD, P, CEC, VA	Cephalosporins (FEP, CEC), Carbapenems (IPM), Tetracyclines (TE), Rifamycins (RD), Penicillins (P), Glycopeptides (VA)	6	0.412		
	Sausage sandwiches (*n* = 16)	FEP, IPM, TE, P, AMX, VA, CL	Cephalosporins (FEP, CL), Carbapenems (IPM), Tetracyclines (TE), Penicillins (P, AMX), Glycopeptides (VA)	5	0.412		
	Luncheon slices (*n* = 19)	FEP, IPM, TE, RD, VA	Cephalosporins (FEP), Carbapenems (IPM), Tetracyclines (TE), Rifamycins (RD), Glycopeptides (VA)	5	0.294		
	Luncheon slices (*n* = 16)	FEP, IPM, P, VA	Cephalosporins (FEP), Carbapenems (IPM), Penicillins (P), Glycopeptides (VA)	4	0.235		
	Luncheon slices (*n* = 14)	FEP, IPM, TE	Cephalosporins (FEP), Carbapenems (IPM), Tetracyclines (TE)	3	0.176		
Sum 148		Average MAR Index for *E. faecalis*			0.461		
*E. faecium*	Sausage sandwiches (*n* = 26)	FEP, IPM, TE, RD, P, CEC, AMX, SXT, VA, AZM, CL, NA, CN, CIP, LEV, LZD, C	Cephalosporins (FEP, CEC, CL), Carbapenems (IPM), Tetracyclines (TE), Rifamycins (RD), Penicillins (P, AMX), Sulfonamides (SXT), Glycopeptides (VA), Macrolides (AZM), Quinolones (NA, CIP, LEV), Aminoglycosides (CN), Oxazolidinones (LZD), Phenicols (C)	12	1	Pandrug-resistant	27 (7.4%)
	Sausage sandwich (*n* = 1)	FEP, IPM, TE, RD, P, CEC, AMX, SXT, VA, AZM, CL, NA, CN, LEV, LZD, C	Cephalosporins (FEP, CEC, CL), Carbapenems (IPM), Tetracyclines (TE), Rifamycins (RD), Penicillins (P, AMX), Sulfonamides (SXT), Glycopeptides (VA), Macrolides (AZM), Quinolones (NA, LEV), Aminoglycosides (CN), Oxazolidinones (LZD), Phenicols (C)	12	0.941		
	Luncheon slices (*n* = 44)	FEP, IPM, TE, RD, P, CEC, AMX, SXT, AZM, CL, NA, CN, CIP, LEV, LZD	Cephalosporins (FEP, CEC, CL), Carbapenems (IPM), Tetracyclines (TE), Rifamycins (RD), Penicillins (P, AMX), Sulfonamides (SXT), Macrolides (AZM), Quinolones (NA, CIP, LEV), Aminoglycosides (CN), Oxazolidinones (LZD)	11	0.882	Extensively drug-resistant	47 (12.8%)
	Sausage sandwiches (*n* = 1)	FEP, IPM, TE, RD, P, CEC, SXT, VA, AZM, CL, NA, CN, LEV, C	Cephalosporins (FEP, CEC, CL), Carbapenems (IPM), Tetracyclines (TE), Rifamycins (RD), Penicillins (P), Sulfonamides (SXT), Glycopeptides (VA), Macrolides (AZM), Quinolones (NA, LEV), Aminoglycosides (CN), Phenicols (C)	11	0.823		
	Luncheon slices (*n* = 2)	FEP, IPM, TE, RD, P, SXT, AZM, CL, NA, CIP, LEV, LZD	Cephalosporins (CL), Carbapenems (IPM), Tetracyclines (TE), Rifamycins (RD), Penicillins (P), Sulfonamides (SXT), Macrolides (AZM), Quinolones (NA, CIP, LEV), Oxazolidinones (LZD)	10	0.705		
	Luncheon slices (*n* = 27)	FEP, IPM, TE, RD, P, CEC, AMX, SXT, AZM, CL, NA, CN, LEV	Cephalosporins (FEP, CEC, CL), Carbapenems (IPM), Tetracyclines (TE), Rifamycins (RD), Penicillins (P, AMX), Sulfonamides (SXT), Macrolides (AZM), Quinolones (NA, LEV), Aminoglycosides (CN)	9	0.765	Multidrug-resistant	292 (79.8%)
	Sausage sandwiches (*n* = 2)	FEP, IPM, TE, RD, P, CEC, SXT, VA, AZM, NA, CL	Cephalosporins (FEP, CEC, CL), Carbapenems (IPM), Tetracyclines (TE), Rifamycins (RD), Penicillins (P), Sulfonamides (SXT), Glycopeptides (VA), Macrolides (AZM), Quinolones (NA)	9	0.647		
	Luncheon slices (*n* = 26)	FEP, IPM, TE, RD, P, CEC, AMX, VA, AZM, NA, CN, CIP	Cephalosporins (FEP, CEC), Carbapenems (IPM), Tetracyclines (TE), Rifamycins (RD), Penicillins (P, AMX), Glycopeptides (VA), Macrolides (AZM), Quinolones (NA, CIP), Aminoglycosides (CN)	10	0.705		
	Sausage sandwiches (*n* = 45)	FEP, IPM, TE, RD, P, CEC, AMX, SXT, VA, AZM, CL	Cephalosporins (FEP, CEC, CL), Carbapenems (IPM), Tetracyclines (TE), Rifamycins (RD), Penicillins (P, AMX), Sulfonamides (SXT), Glycopeptides (VA), Macrolides (AZM)	8	0.647		
	Sausage sandwiches (*n* = 40)	FEP, IPM, TE, RD, P, CEC, AMX, SXT, VA	Cephalosporins (FEP, CEC), Carbapenems (IPM), Tetracyclines (TE), Rifamycins (RD), Penicillins (P, AMX), Sulfonamides (SXT), Glycopeptides (VA)	7	0.529		
	Luncheon slices (*n* = 53)	FEP, IPM, TE, RD, P, CEC, SXT, VA	Cephalosporins (FEP, CEC), Carbapenems (IPM), Tetracyclines (TE), Rifamycins (RD), Penicillins (P), Sulfonamides (SXT), Glycopeptides (VA)	7	0.471		
	Sausage sandwiches (*n* = 43)	FEP, IPM, TE, RD, P, CEC, SXT	Cephalosporins (FEP, CEC), Carbapenems (IPM), Tetracyclines (TE), Rifamycins (RD), Penicillins (P), Sulfonamides (SXT)	6	0.412		
	Sausage sandwich (*n* = 2)	FEP, IPM, TE, RD, P, CEC, VA	Cephalosporins (FEP, CEC), Carbapenems (IPM), Tetracyclines (TE), Rifamycins (RD), Penicillins (P), Glycopeptides (VA)	6	0.412		
	Sausage sandwiches (*n* = 31)	FEP, IPM, TE, RD, P, AZM	Cephalosporins (FEP), Carbapenems (IPM), Tetracyclines (TE), Rifamycins (RD), Penicillins (P), Macrolides (AZM)	6	0.353		
	Luncheon slices (*n* = 23)	FEP, IPM, TE, RD, VA	Cephalosporins (FEP), Carbapenems (IPM), Tetracyclines (TE), Rifamycins (RD), Glycopeptides (VA)	5	0.294		
Sum 366		Average MAR Index for *E. faecium*			0.600		

Legend: C, Chloramphenicol; FEP, Cefepime; P, Penicillin; IMP, Imipenem; VA, Vancomycin; RD, Rifampin; CEC, Cefaclor; SXT, Trimethoprim/Sulfamethoxazole; TE, Tetracycline; AZM, Azithromycin; CL, Cephalexin; LZD, Linezolid; CIP, Ciprofloxacin; CN, Gentamicin; LEV, Levofloxacin; NA, Nalidixic acid; AMX, Amoxicillin. * Classification of strains into multidrug-resistant (MDR), extensively drug-resistant (XDR), and pandrug-resistant (PDR) was applied according to the international standard proposed by Magiorakos et al. [48]. MDR was defined as acquired non-susceptibility to at least one agent in three or more antimicrobial categories. XDR was defined as non-susceptibility to at least one agent in all but two or fewer antimicrobial categories (i.e., isolates remain susceptible to only one or two categories). PDR was defined as non-susceptibility to all agents in all antimicrobial categories tested. The 17 antibiotics tested represented 12 different antimicrobial classes, allowing classification according to these criteria.

### 3.6. Prevalence of Vancomycin Resistance Genes Among MDR Enterococci Isolates

**Figure 6:**
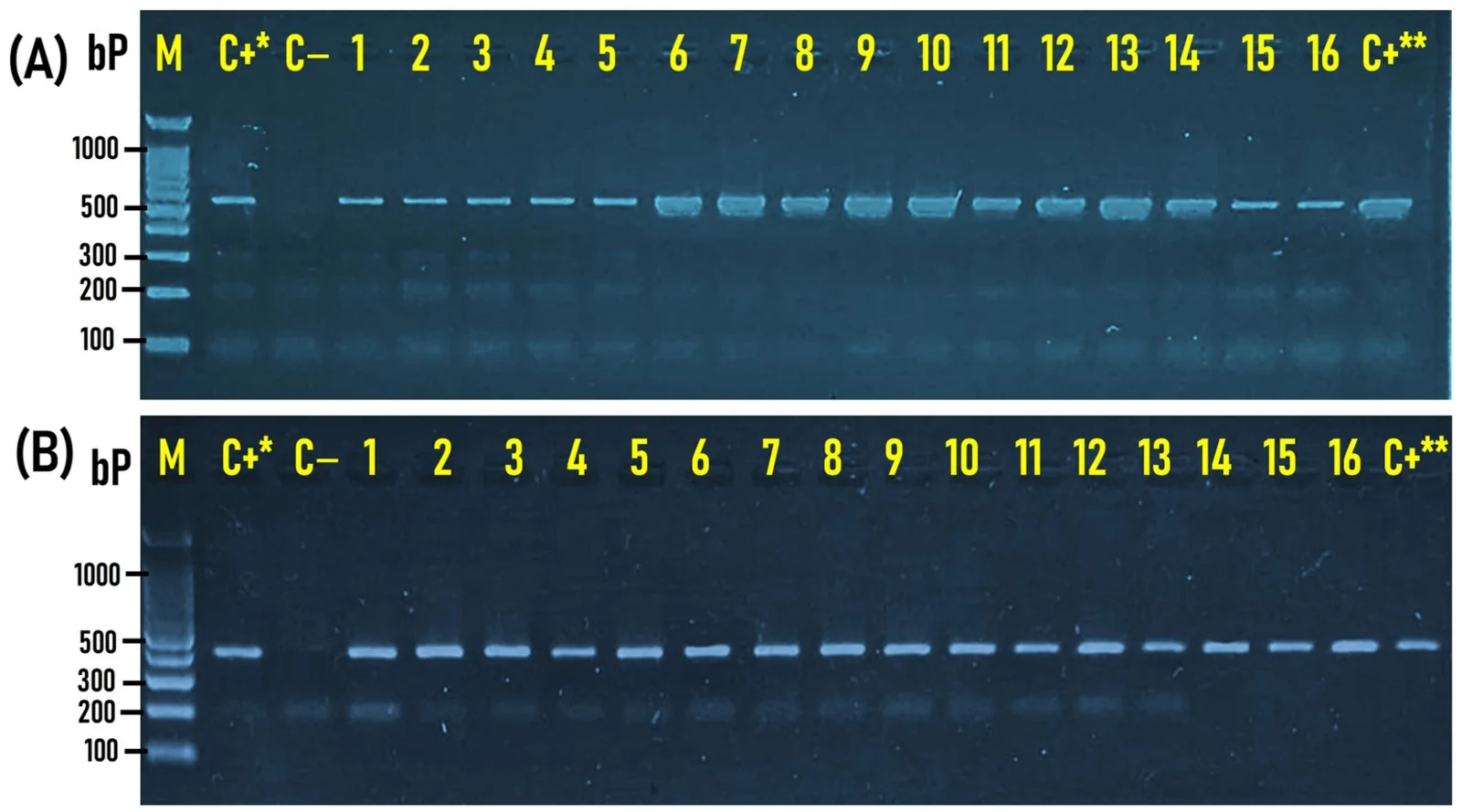
Detection of vancomycin resistance genes in *Enterococcus* isolates. Agarose gel electrophoresis of PCR products targeting the *vanA* (**A**) and *vanB* (**B**) genes is shown. Lanes 1–8 represent *Enterococcus faecalis* isolates, while lanes 9–16 correspond to *Enterococcus faecium* isolates obtained from ready-to-eat (RTE) meat samples. Amplified fragments were observed at the expected sizes of 559 bp for the *vanA* gene (**A**) and 467 bp for the *vanB* gene (**B**). M: 100 bp DNA ladder. Positive controls included *E. faecalis* ATCC 29212 (C+*) and *E. faecium* ATCC 19434 (C+**), whereas Escherichia coli K-12 DH5α served as the negative control (C−). PCR products (8 µL) were resolved on a 1.5% agarose gel and visualized under ultraviolet light.

In the present study, the PCR technique targeted the vancomycin resistance genes *vanA* and *vanB* in *E. faecalis* (*n* = 148) and *E. faecium* (*n* = 366) isolates recovered from RTE meat products, and the PCR products were amplified at the expected molecular sizes of 559 bp (Figure 6A) and 467 bp (Figure 6B), respectively. Molecular analysis by polymerase chain reaction (PCR) confirmed the presence of *vanA* and/or *vanB* genes, either individually or in combination, in all of the 321 phenotypically vancomycin-resistant *Enterococcus* (VRE) isolates. A collective dataset showing the antimicrobial resistance profile in conjunction with the molecular characterization of *Enterococcus* isolates (*n* = 514) isolated from RTE luncheon slices and sausage sandwiches is demonstrated in Table 4.

In the present study, complete concordance was observed between phenotypic vancomycin resistance and detection of *vanA*/*vanB*, as no van-gene-positive isolate was phenotypically susceptible to vancomycin. Although genotype–phenotype discrepancies can occur because the presence of a resistance determinant does not invariably indicate its phenotypic expression, such discordance was not observed in the isolates examined here. This may partly reflect the phenotypic selection of isolates subjected to molecular confirmation and the expression of the detected resistance determinants under the conditions used in this study.

The *vanA* gene was the predominant resistance determinant, followed by *vanB*. Of the 321 VRE (102 *E. faecalis* and 219 *E. faecium*) isolates examined, 189 (60.6%) carried the *vanA* gene alone, including 99 isolates from luncheon meat and 90 from sausage samples. In contrast, only 28 isolates (5.45%) were positive for the *vanB* gene alone, with 13 originating from luncheon and 15 from sausage. Additionally, 104 isolates (20.2%) harbored both *vanA* and *vanB* genes, distributed as 40 and 64 isolates from luncheon and sausage, respectively (Table 1 and Table 4). On the species basis, 72.5% (74/102) of vancomycin-resistant *E. faecalis* isolates harbored the *vanA* gene, while only 5.9% (6/102) of such isolates harbored the *vanB* gene alone, whereas 21.6% (22/102) of the isolates showed coexistence of both *vanA* and *vanB* genes. On the other hand, *vanA*, *vanB*, and the co-occurrence of both *vanA* and *vanB genes* were detected in 52.5% (115/219), 10.05% (22/219), and 37.4% (82/219) of vancomycin-resistant *E. faecium* isolates (Table 1 and Table 4).

The present study revealed a high prevalence of vancomycin resistance genes among VRE isolates from luncheon and sausage products, with *vanA* being the dominant gene. This pattern agrees with previous studies identifying *vanA* as the most prevalent vancomycin resistance determinant in foodborne *Enterococcus* spp. Similar findings were reported in Canada, where *vanA* was found in over 60% of isolates from retail meats [18]. Additionally, the coexistence of *vanA* and *vanB* in 20.2% of isolates in the present study is comparable with observations reported by Torres et al. [49], who highlighted the role of horizontal gene transfer in food-associated *Enterococcus* spp. Overall, the high frequency of vancomycin resistance genes detected in this study is comparable to global reports and underscores the public health risk associated with processed meat products as potential reservoirs of VRE. 

### 3.7. Effect of Extra Heat Treatment of RTE Sausage and Luncheon on Survival of Enterococcus spp.

**Figure 7:**
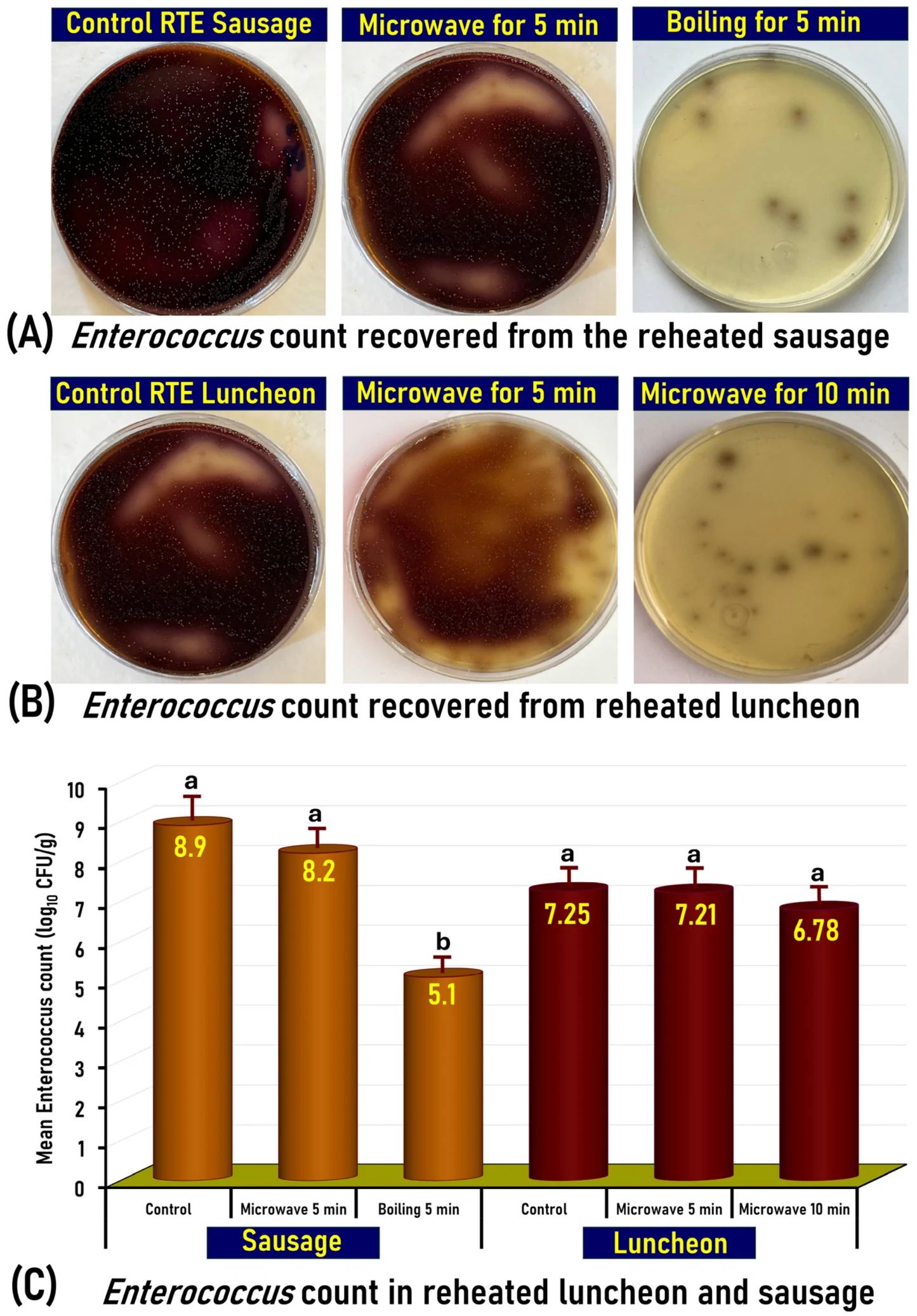
Effect of post-purchase reheating treatments on the survival of naturally occurring *Enterococcus* spp. in ready-to-eat (RTE) sausage and luncheon products. (**A**) Representative Bile Esculin Azide agar plates (10^−4^ dilution) showing *Enterococcus* recovery from RTE sausage before reheating (control), after microwave heating for 5 min, and after boiling for 5 min. (**B**) Representative Bile Esculin Azide agar plates (10^−3^ dilution) showing *Enterococcus* recovery from RTE luncheon before reheating (control), after microwave heating for 5 min, and after microwave heating for 10 min. (**C**) Mean *Enterococcus* counts (log_10_ CFU/g) recovered from reheated sausage and luncheon samples. Data are presented as mean ± SD (*n* = 9) of three independent trials. Different lowercase letters above bars indicate significant differences among treatments within the same product category (*p* < 0.05; one-way ANOVA followed by Tukey’s post hoc test).

The effects of additional heat treatments on *Enterococcus* counts in ready-to-eat (RTE) meat products are shown in Figure 7. Although the luncheon slices and sausage sandwiches tested are ready-to-eat meat products that require no heating before consumption, an experiment was applied in the current study to estimate the capability of *Enterococcus* to survive extra heat treatments.

Boiling completely immersed sausage samples for 5 min with an internal core temperature of approximately 85 °C resulted in a marked, statistically significant reduction (*p* < 0.01) of approximately 3.8 log cycles in enterococcal counts (8.9 ± 0.52 vs. 5.1 ± 0.25 log_10_ CFU/g), representing a reduction of about 99.98% in viable counts (Figure 7A,C). The measured core temperature of approximately 85 °C confirms that the treatment resulted in substantial heating of the sausage interior; therefore, the residual survival was not simply attributable to failure of heat to reach the product core. Nevertheless, the present experiment was not designed to determine strain-specific thermal resistance or thermal death kinetics. The residual survival may reflect differences in the thermal tolerance of the enterococcal strains and/or protective effects of the meat matrix. Further studies incorporating controlled time–temperature profiles and determination of D- and z-values would be required to characterize the thermal resistance of the recovered strains.

Amazingly, microwave treatment (220 V, 2450 MHz, 1200 W) of sausage samples for 5 min produced a limited reduction of approximately 0.7 log cycles (8.9 ± 0.52 vs. 8.2 ± 0.45 log_10_ CFU/g), which was not statistically significant (*p* > 0.05). Similarly, microwave heating of beef luncheon samples for 5 and 10 min resulted in minimal reductions in *Enterococcus* counts. Mean counts decreased from 7.25 ± 0.42 log_10_ CFU/g in the control to 7.21 ± 0.35 and 6.78 ± 0.38 log_10_ CFU/g after 5 and 10 min of treatment, corresponding to reductions of approximately 0.04 and 0.47 log cycles, respectively. These reductions were not statistically significant (*p* > 0.05). (Figure 7B,C).

Overall, microwave heating under the applied conditions resulted in minor reductions in enterococcal counts in both luncheon and sausage samples. However, because internal product temperatures were not monitored, the observed reductions cannot be directly related to the thermal exposure achieved within the samples. A limitation of the microwave-heating experiment was that the internal temperature of the luncheon and sausage samples was not measured during or immediately after treatment. Consequently, the relatively minor reduction in enterococcal counts cannot be directly related to the temperature attained within the food matrix. Microwave heating may produce spatially heterogeneous temperature distributions, and differences in product composition and matrix structure may further affect heat transfer and microbial inactivation. Therefore, the observed reductions should be interpreted as treatment-specific microbiological effects rather than as evidence of a defined thermal inactivation response. Future studies should monitor internal temperatures during microwave treatment to establish the actual thermal exposure and its relationship with enterococcal reduction. On the other hand, boiling resulted in a substantial, although incomplete, reduction, highlighting the notable heat tolerance of these organisms in RTE meat matrices.

Furthermore, 13 *Enterococcus* isolates (3 *E. faecalis* and 10 *Enterococcus faecium*), which passed the extra heat treatment from both products, were randomly selected and tested for antibiotic resistance, as well as for the presence of the virulence and resistance genes (Table 5). Interestingly, all (100%) of the 13 enterococcal isolates harbored both the *gelE* and *ace* genes. Moreover, 11 of the 13 (84.6%) isolates were VRE, harboring *vanA* (6/11; 54.6%), *vanB* (1/13, 7.7%), or both *vanA* and *vanB* genes together (4/11; 36.4%) (Table 5). Notably, 1 (7.7%), 2 (15.4%), and 10 (76.9%) of such thermotolerant enterococcal isolates were classified as pandrug-resistant, extensively drug-resistant, and multidrug-resistant, respectively (Table 5).

While all 13 heat-surviving isolates retained the *gelE* and *ace* virulence genes, our assessment was limited to these two targets and did not include screening for other virulence determinants (e.g., *asa1*, *cylA*, *esp*, *hyl*) or whole-genome sequencing. Consequently, we cannot exclude the possibility that heat stress may have influenced the presence or expression of untested virulence genes. Nevertheless, the consistent retention of both *gelE* and *ace* suggests that these clinically relevant virulence factors are not readily lost following sublethal heat exposure. Future studies employing comprehensive genomic approaches are warranted to fully elucidate the impact of thermal stress on the virulence potential of foodborne enterococci.

These findings highlight the serious public health risk posed by *Enterococcus faecalis* and *E. faecium*, which can survive common food processing methods such as boiling and microwave heating. Their multidrug resistance and possession of multiple virulence genes, combined with their persistence in ready-to-eat meats, reveal gaps in current food safety measures. This resilience complicates infection control and limits treatment options, emphasizing the need for improved control strategies and alternative therapies.

From a food safety perspective, these findings emphasize that commonly applied reheating practices, particularly microwave heating, may be insufficient to control thermotolerant enterococci in RTE meat products. Therefore, stricter control measures, including improved processing hygiene, optimized thermal treatments, and consumer awareness regarding effective reheating methods, are essential to mitigate potential public health risks.

## 4. Conclusions

In conclusion, this study demonstrates widespread contamination of ready-to-eat (RTE) beef luncheon and sausage with *Enterococcus faecalis* and *Enterococcus faecium*, with *E. faecium* being more common. The isolates showed a high prevalence of virulence factors, especially *gelE* and *ace*, along with concerning levels of antimicrobial resistance, including multidrug-, extensively drug-, and pandrug-resistant types. The frequent presence of vancomycin resistance genes (*vanA* and *vanB*) highlights the clinical importance of these strains as potential sources of last-resort antibiotic resistance. Notably, the ability of enterococci to survive additional heat treatments, particularly microwave exposure, emphasizes their significant heat tolerance and persistence in RTE meat products. The combination of heat resistance, antimicrobial resistance, and virulence traits complicates both food safety efforts and clinical management of infections. Therefore, strict hygienic practices during processing, ongoing surveillance of antimicrobial resistance, and responsible antibiotic use in food animal production are vital. Additionally, developing more effective intervention strategies and alternative control measures is urgently needed to reduce the spread of these resilient pathogens.

Overall, these results suggest that RTE meats may serve as potential reservoirs of highly resistant and virulent enterococci. While direct epidemiological evidence linking these foodborne isolates to clinical infections was not established, their presence in ready-to-eat products warrants public health attention. Further epidemiological and transmission studies are needed to determine the actual clinical significance of foodborne enterococci and to guide evidence-based interventions.

## Figures and Tables

**Table 1:** Distribution of virulence and antimicrobial resistance genes in *E. faecalis* (*n* = 148) and *E. faecium* (*n* = 366) isolates from luncheon slices and sausage sandwiches.

*Enterococcus* spp.	Meat Products	Number of Isolates	Virulence Genes			Resistance Gene		
			*sodA*	*gelE*	*ace*	*vanA*	*vanB*	*vanA* + *vanB*
*E. faecalis*	Luncheon slices	88	88 (100%)	88 (100%)	43 (48.9%)	38 (43.2%)	1 (1.14%)	11 (12.5%)
	Sausage sandwiches	60	60 (100%)	60 (100%)	41 (68.3%)	36 (60%)	5 (8.3%)	11 (18.3%)
	Total	148	148 (100%)	148 (100%)	84 (56.8%)	74 (50%)	6 (4.05%)	22 (14.9%)
*E. faecium*	Luncheon slices	175	175 (100%)	153 (87.4%)	107 (61.1%)	61 (34.9%)	12 (6.9%)	29 (16.6%)
	Sausage sandwiches	191	191 (100%)	191 (100%)	121 (63.4%)	54 (28.3%)	10 (5.24%)	53 (27.7%)
	Total	366	366 (100%)	344 (94%)	228 (62.3%)	115 (31.4%)	22 (6.01%)	82 (22.4%)
Overall		514	514 (100%)	492 (95.7%)	312 (60.7%)	189 (36.8%)	28 (5.45%)	104 (20.2%)

Legend: *sodA*: superoxide dismutase gene (species-specific marker); *gelE*: gelatinase gene (virulence factor); *ace*: collagen-binding protein gene (virulence factor); *vanA* and *vanB*: vancomycin resistance genes. *n*: Number of isolates; %: Percentage of positive isolates.

**Table 2:** Antibiotic resistance profile of *enterococci* isolates recovered from luncheon slices and sausage sandwiches against 17 antimicrobials.

*Enterococcus* Sources	Isolates	Antimicrobials																
		C	CL	CEC	CIP	SXT	FEP	LZD	LEV	AZM	IMP	RD	VA	NA	CN	P	TE	Amx
Luncheon slices	*E. faecalis* (*n* = 88)	24 (27.3%)	37 (42%)	39 (44.3%)	0 (0%)	38 (43.2%)	88 (100%)	0 (0%)	0 (0%)	21 (23.9%)	88 (100%)	58 (65.9%)	50 (56.8%)	24 (27.3%)	0 (0%)	55 (62.5%)	72 (81.8%)	26 (29.5%)
	*E. faecium* (*n* = 175)	0 (0%)	73 (41.7%)	150 (85.7%)	72 (41.1%)	126 (72%)	175 (100%)	46 (26.3%)	73 (41.7%)	99 (56.6%)	175 (100%)	175 (100%)	102 (58.3%)	99 (56.6%)	97 (55.4%)	152 (86.9%)	175 (100%)	97 (55.4%)
	Sum (*n* = 263)	24 (9.1%)	110 (41.8%)	189 (71.9%)	72 (27.4%)	164 (62.4%)	263 (100%)	46 (17.5%)	73 (27.8%)	120 (45.6%)	263 (100%)	233 (88.6%)	152 (57.8%)	123 (46.8%)	97 (36.9%)	207 (78.7%)	247 (93.9%)	123 (46.8%)
Sausage sandwiches	*E. faecalis* (*n* = 60)	20 (33.3%)	36 (60%)	42 (70%)	20 (33.3%)	44 (73.3%)	60 (100%)	12 (20%)	20 (33.3%)	8 (13.3%)	60 (100%)	44 (73.3%)	52 (86.7%)	0 (0%)	0 (0%)	60 (100%)	45 (75%)	18 (30%)
	*E. faecium* (*n* = 191)	28 (14.7%)	75 (39.3%)	160 (83.8%)	26 (13.6%)	158 (82.7%)	191 (100%)	27 (14.1%)	28 (14.7%)	106 (55.5%)	191 (100%)	191 (100%)	117 (61.3%)	30 (15.7%)	28 (14.7%)	191 (100%)	191 (100%)	112 (58.6%)
	Sum (*n* = 251)	48 (19.1%)	111 (44.2%)	202 (80.5%)	46 (18.3%)	202 (80.5%)	251 (100%)	39 (15.5%)	48 (19.1%)	114 (45.4%)	251 (100%)	235 (93.6%)	169 (67.3%)	30 (12%)	28 (11.2%)	251 (100%)	236 (94%)	130 (51.8%)
Sum	*E. faecalis* (*n* = 148)	44 (29.7%)	73 (49.3%)	81 (54.7%)	20 (13.5%)	82 (55.4%)	148 (100%)	12 (8.1%)	20 (13.5%)	29 (19.6%)	148 (100%)	102 (68.9%)	102 (68.9%)	24 (16.2%)	0 (0%)	115 (77.7%)	117 (79.1%)	44 (29.7%)
	*E. faecium* (*n* = 366)	28 (7.7%)	148 (40.4%)	310 (84.7%)	98 (26.8%)	284 (77.6%)	366 (100%)	73 (19.9%)	101 (27.6%)	205 (56%)	366 (100%)	366 (100%)	219 (59.8%)	129 (35.2%)	125 (34.2%)	343 (93.7%)	366 (100%)	209 (57.1%)
Overall resistance (*n* = 514)		72 (14%)	221 (43%)	391 (76.1%)	118 (23%)	366 (71.2%)	514 (100%)	85 (16.5%)	121 (23.5%)	234 (45.5%)	514 (100%)	468 (91%)	321 (62.5%)	153 (29.8%)	125 (24.3%)	458 (89.1%)	483 (94%)	253 (50.2%)

Legend: C, Chloramphenicol (30 µg); CL, Cephalexin (30 µg); CEC, Cefaclor (30 µg); CIP, Ciprofloxacin (5 µg); SXT, Trimethoprim-Sulfamethoxazole (25 µg); FEP, Cefepime (30 µg); LZD, Linezolid (10 µg); LEV, Levofloxacin (5 µg); AZM, Azithromycin (15 µg); IMP, Imipenem (10 µg); RD, Rifampin (30/10 µg); VA, Vancomycin (30 µg); NA, Nalidixic acid (30 µg); CN, Gentamicin (10 µg); P, Penicillin G (50 µg); TE, Tetracycline (30 µg); AMX, Amoxicillin (10 µg). Data are presented as the number of resistant isolates with percentages in parentheses.

**Table 4:** Antimicrobial resistance profile and molecular characterization of *Enterococcus* isolates (*n* = 514) isolated from luncheon slices and sausage sandwiches.

*Enterococcus* spp.	Source	No. of Isolates	Resistance Profile	Virulence Gene	Resistance Genes
*E. faecalis* (*n* = 148)	Luncheon slices	13	FEP, IPM, TE, RD, P, CEC, AMX, SXT, AZM, CL, NA, C	*sodA*, *gelE*, *ace*	*-*
		8	FEP, IPM, TE, RD, P, CEC, AMX, SXT, AZM, CL, NA, C	*sodA*, *gelE*	*-*
		3	FEP, IPM, TE, RD, P, CEC, AMX, SXT, CL, NA, C	*sodA*, *gelE*	*-*
		13	FEP, IPM, TE, RD, P, CEC, SXT, VA, CL	*sodA*, *gelE*	*vanA*
		1	FEP, IPM, TE, RD, P, CEC, AMX, SXT, VA	*sodA*, *gelE*	*vanA*
		1	FEP, IPM, TE, RD, P, AMX, CEC, VA	*sodA*, *gelE*	*vanB*
		7	FEP, IPM, TE, RD, VA	*sodA*, *gelE*	*vanA*, *vanB*
		12	FEP, IPM, TE, RD, VA	*sodA*, *gelE*	*vanA*
		12	FEP, IPM, P, VA	*sodA*, *gelE*, *ace*	*vanA*
		4	FEP, IPM, P, VA	*sodA*, *gelE*, *ace*	*vanA*, *vanB*
		14	FEP, IPM, TE	*sodA*, *gelE*, *ace*	-
	Sausage sandwiches	5	FEP, IPM, TE, RD, P, CEC, SXT, AMX, VA, CL, CIP, LEV, LZD, C	*sodA*, *gelE*, *ace*	*vanA*, *vanB*
		3	FEP, IPM, TE, RD, P, CEC, SXT, AMX, VA, CL, CIP, LEV, LZD, C	*sodA*, *gelE*, *ace*	*vanA*
		4	FEP, IPM, TE, RD, P, CEC, SXT, AMX, VA, CL, CIP, LEV, LZD, C	*sodA*, *gelE*	*vanA*
		6	FEP, IPM, TE, RD, P, CEC, SXT, AMX, AZM, CL, CIP, LEV, C	*sodA*, *gelE*, *ace*	-
		2	FEP, IPM, TE, RD, P, SXT, AZM, CL, CIP, LEV, C	*sodA*, *gelE*, *ace*	-
		9	FEP, IPM, TE, P, CEC, SXT, VA, RD	*sodA*, *gelE*, *ace*	*vanA*
		10	FEP, IPM, RD, P, CEC, SXT, VA	*sodA*, *gelE*	*vanA*
		5	FEP, IPM, RD, P, CEC, SXT, VA	*sodA*, *gelE*	*vanB*
		10	FEP, IPM, TE, P, VA, CL	*sodA*, *gelE*, *ace*	*vanA*
		6	FEP, IPM, TE, P, VA, CL	*sodA*, *gelE*, *ace*	*vanA*, *vanB*
*E. faecium* (*n* = 366)	Luncheon slices	30	FEP, IPM, TE, RD, P, CEC, AMX, SXT, AZM, CL, NA, CN, CIP, LEV, LZD	*sodA, gelE, ace*	-
		14	FEP, IPM, TE, RD, P, CEC, SXT, AZM, CL, NA, CN, CIP, LEV, LZD	*sodA*, *gelE*	-
		22	FEP, IPM, TE, RD, P, CEC, AMX, SXT, AZM, CL, NA, CN, LEV	*sodA*	-
		5	FEP, IPM, TE, RD, P, CEC, AMX, SXT, AZM, CL, NA, CN, LEV	*sodA*, *gelE*	-
		2	FEP, IPM, TE, RD, P, SXT, AMX, AZM, CL, NA, CIP, LEV, LZD	*sodA*, *gelE*, *ace*	-
		9	FEP, IPM, TE, RD, P, CEC, AMX, VA, AZM, NA, CN, CIP	*sodA*, *gelE*, *ace*	*vanA*, *vanB*
		17	FEP, IPM, TE, RD, P, CEC, AMX, VA, AZM, NA, CN, CIP	*sodA*, *gelE*, *ace*	*vanA*
		12	FEP, IPM, TE, RD, P, CEC, AMX, SXT, VA	*sodA*, *gelE*, *ace*	*vanA*, *vanB*
		14	FEP, IPM, TE, RD, P, CEC, SXT, VA	*sodA*, *gelE*, *ace*	*vanA*
		14	FEP, IPM, TE, RD, P, CEC, SXT, VA	*sodA*, *gelE*	*vanA*
		5	FEP, IPM, TE, RD, P, CEC, SXT, VA	*sodA*, *gelE*	*vanB*
		8	FEP, IPM, TE, RD, P, CEC, SXT, VA	*sodA*, *gelE*	*vanA*, *vanB*
		7	FEP, IPM, TE, RD, VA	*sodA*, *gelE*, *ace*	*vanB*
		16	FEP, IPM, TE, RD, VA	*sodA*, *gelE*, *ace*	*vanA*
	Sausage sandwiches	14	FEP, IPM, TE, RD, P, CEC, AMX, SXT, VA, AZM, CL, NA, CN, CIP, LEV, LZD, C	*sodA*, *gelE*, *ace*	*vanA*, *vanB*
		12	FEP, IPM, TE, RD, P, CEC, AMX, SXT, VA, AZM, CL, NA, CN, CIP, LEV, LZD, C	*sodA*, *gelE*, *ace*	*vanA*
		1	FEP, IPM, TE, RD, P, CEC, AMX, SXT, VA, AZM, CL, NA, CN, LEV, LZD, C	*sodA*, *gelE*, *ace*	*vanA*
		1	FEP, IPM, TE, RD, P, CEC, AMX, SXT, VA, AZM, CL, NA, CN, LEV, C	*sodA*, *gelE*, *ace*	*vanA*
		2	FEP, IPM, TE, RD, P, CEC, SXT, VA, AZM, NA, CL	*sodA*, *gelE*	*vanA*, *vanB*
		12	FEP, IPM, TE, RD, P, CEC, AMX, SXT, VA, AZM, CL	*sodA*, *gelE*, *ace*	*vanA*, *vanB*
		12	FEP, IPM, TE, RD, P, CEC, AMX, SXT, VA, AZM, CL	*sodA*, *gelE*, *ace*	*vanA*
		6	FEP, IPM, TE, RD, P, CEC, SXT, VA, AZM, CL	*sodA*, *gelE*, *ace*	*vanB*
		7	FEP, IPM, TE, RD, P, CEC, SXT, VA, AZM, CL	*sodA*, *gelE*	*vanA*
		8	FEP, IPM, TE, RD, P, CEC, SXT, VA, AZM, CL	*sodA*, *gelE*	*vanA*, *vanB*
		8	FEP, IPM, TE, RD, P, CEC, SXT, VA	*sodA*, *gelE*, *ace*	*vanA*, *vanB*
		10	FEP, IPM, TE, RD, P, CEC, AMX, SXT, VA	*sodA*, *gelE*, *ace*	*vanA*
		4	FEP, IPM, TE, RD, P, CEC, AMX, SXT, VA	*sodA*, *gelE*, *ace*	*vanA*
		9	FEP, IPM, TE, RD, P, CEC, AMX, SXT, VA	*sodA*, *gelE*	*vanA*, *vanB*
		5	FEP, IPM, TE, RD, P, CEC, AMX, SXT, VA	*sodA*, *gelE*	*vanA*
		4	FEP, IPM, TE, RD, P, CEC, AMX, SXT, VA	*sodA*, *gelE*	*vanB*
		28	FEP, IPM, TE, RD, P, AMX, CEC, SXT	*sodA*, *gelE*, *ace*	-
		15	FEP, IPM, TE, RD, P, CEC, SXT	*sodA*, *gelE*	-
		2	FEP, IPM, TE, RD, P, CEC, VA	*sodA*, *gelE*	*vanA*
		18	FEP, IPM, TE, RD, P, AZM	*sodA*, *gelE*	-
		13	FEP, IPM, TE, RD, P, AZM	*sodA*, *gelE*, *ace*	-

Legend: C, Chloramphenicol; CL, Cephalexin; CEC, Cefaclor; CIP, Ciprofloxacin; SXT, Trimethoprim/Sulfamethoxazole; FEP, Cefepime; LZD, Linezolid; LEV, Levofloxacin; AZM, Azithromycin; IMP, Imipenem; RD, Rifampin; VA, Vancomycin; NA, Nalidixic acid; CN, Gentamicin; P, Penicillin; TE, Tetracycline; AMX, Amoxicillin.

**Table 5:** Antimicrobial resistance profile and molecular characterization of *Enterococcus* isolates (*n* = 13) recovered from RTE meat products after extra heat treatment.

Type of Heat Processing	Meat Product Type	Isolate spp.	Antimicrobial Resistance Pattern	MAR Index	Isolates Profile	Virulence Genes	Resistance Gene
Microwave	Luncheon slices	*E. faecium*	FEP, IPM, TE, RD, P, AMX, SXT, AZM, CL, NA, CIP, LEV, LZD	0.765	MDR	*sodA*, *gelE*, *ace*	-
		*E. faecium*	FEP, IPM, TE, RD, P, CEC, AMX, VA, AZM, NA, CN, CIP	0.706	MDR	*sodA*, *gelE*, *ace*	*vanA*, *vanB*
		*E. faecalis*	FEP, IPM, TE, RD, P, CEC, SXT, CL, NA, C	0.588	MDR	*sodA*, *gelE*, *ace*	-
		*E. faecium*	FEP, IPM, TE, RD, P, CEC, AMX, SXT, VA	0.529	MDR	*sodA*, *gelE*, *ace*	*vanA*, *vanB*
	Sausage sandwiches	*E. faecium*	FEP, IPM, TE, RD, P, CEC, AMX, SXT, VA, AZM, CL	0.647	MDR	*sodA*, *gelE*, *ace*	*vanA*
		*E. faecium*	FEP, IPM, TE, RD, P, CEC, AMX, SXT, VA, AZM, CL, NA, CN, CIP, LEV, LZD, C	1.0	PDR	*sodA*, *gelE*, *ace*	*vanA*
		*E. faecalis*	FEP, IPM, TE, RD, P, CEC, AMX, SXT, VA, CL, CIP, LEV, LZD, C	0.823	XDR	*sodA*, *gelE*, *ace*	*vanA*
		*E. faecalis*	FEP, IPM, TE, P, CEC, SXT, VA, RD	0.471	MDR	*sodA*, *gelE*, *ace*	*vanA*
Boiling	Sausage sandwiches	*E. faecium*	FEP, IPM, TE, RD, P, CEC, AMX, SXT, VA, AZM, CL, NA, CN, CIP, LEV, LZD, C	1.0	PDR	*sodA*, *gelE*, *ace*	*vanA*, *vanB*
		*E. faecium*	FEP, IPM, TE, RD, P, CEC, AMX, SXT, VA, AZM, CL, NA, CN, CIP, LEV, LZD, C	1.0	PDR	*sodA*, *gelE*, *ace*	*vanA*, *vanB*
		*E. faecium*	FEP, IPM, TE, RD, P, CEC, AMX, SXT, VA, AZM, CL, NA, CN, LEV, C	0.882	XDR	*sodA*, *gelE*, *ace*	*vanA*
		*E. faecium*	FEP, IPM, TE, RD, P, CEC, SXT, VA, AZM, CL	0.588	MDR	*sodA*, *gelE*, *ace*	*vanA*
		*E. faecium*	FEP, IPM, TE, RD, P, CEC, SXT	0.412	MDR	*sodA*, *gelE*, *ace*	*vanB*

Legend: C, Chloramphenicol; CL, Cephalexin; CEC, Cefaclor; CIP, Ciprofloxacin; SXT, Trimethoprim/Sulfamethoxazole; FEP, Cefepime; LZD, Linezolid; LEV, Levofloxacin; AZM, Azithromycin; IMP, Imipenem; RD, Rifampin; VA, Vancomycin; NA, Nalidixic acid; CN, Gentamicin; P, Penicillin; TE, Tetracycline; AMX, Amoxicillin.

## Data Availability

The original contributions presented in this study are included in the article. Further inquiries can be directed to the corresponding authors.
